# Phylogenetic Analysis of Thecosomata Blainville, 1824 (Holoplanktonic Opisthobranchia) Using Morphological and Molecular Data

**DOI:** 10.1371/journal.pone.0059439

**Published:** 2013-04-12

**Authors:** Emmanuel Corse, Jeannine Rampal, Corinne Cuoc, Nicolas Pech, Yvan Perez, André Gilles

**Affiliations:** IMBE (UMR CNRS 7263, IRD 237) Evolution Génome Environnement, Aix-Marseille Université, Marseille, France; Biodiversity Insitute of Ontario – University of Guelph, Canada

## Abstract

Thecosomata is a marine zooplankton group, which played an important role in the carbonate cycle in oceans due to their shell composition. So far, there is important discrepancy between the previous morphological-based taxonomies, and subsequently the evolutionary history of Thecosomata. In this study, the remarkable planktonic sampling of TARA Oceans expedition associated with a set of various other missions allowed us to assess the phylogenetic relationships of Thecosomata using morphological and molecular data (28 S and COI genes). The two gene trees showed incongruities (e.g. *Hyalocylis, Cavolinia*), and high congruence between morphological and 28S trees (e.g. monophyly of Euthecosomata). The monophyly of straight shell species led us to reviving the Orthoconcha, and the split of Limacinidae led us to the revival of *Embolus inflata* replacing *Limacina inflata*. The results also jeopardized the Euthecosomata families that are based on plesiomorphic character state as in the case for Creseidae which was not a monophyletic group. Divergence times were also estimated, and suggested that the evolutionary history of Thecosomata was characterized by four major diversifying events. By bringing the knowledge of palaeontology, we propose a new evolutionary scenario for which macro-evolution implying morphological innovations were rhythmed by climatic changes and associated species turn-over that spread from the Eocene to Miocene, and were shaped principally by predation and shell buoyancy.

## Introduction

Thecosomata are marine holoplanktonic opistobranch molluscs, which can be found in various depths in all oceans [1]. They are considered as a remarkable model for monitoring the effect of oceans' acidification on calcifying organisms [2], [3], [4], [5], [6]. Belonging to Pteropoda [7], Thecosomata are not only characterized by their foot's modification into a swimming organ (parapodia or swimming parapodial disc) but also by a broad morphological diversity of the shell. So far, different shell types have been described including species with a calcareous coiled shell as exhibited by the last Thecosomata ancestor and species with calcareous or aminated bilaterally symmetrical straight shell more adapted to a planktonic lifestyle [8], [9].

The loss or the morphological diversifying of the shell took place during the transition from a benthic to a pelagic lifestyle and represents important morphogenetic steps during the evolutionary history of Thecosomata. It was argued that this adaptation to pelagic lifestyle resulted from a neotenic process [10], [11].

**Figure 1:**
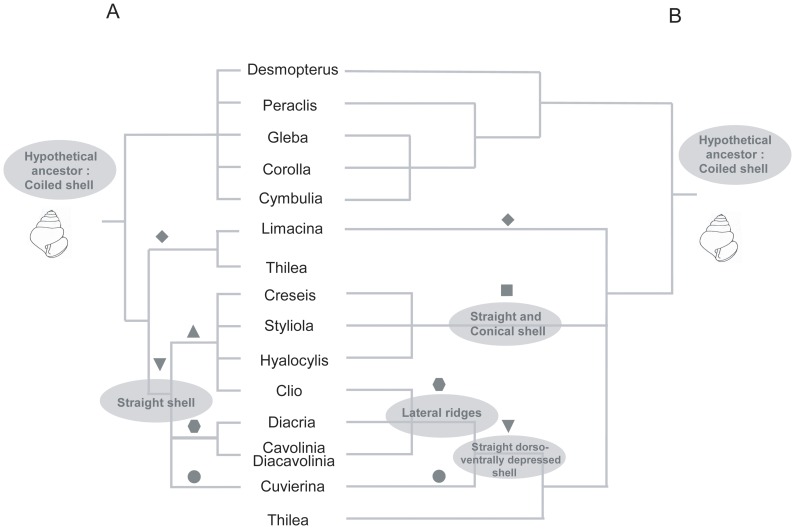
Different phylogenetic hypothesis of Euthecosomata. A) The left topology is deduced from Rampal studies which considered two straight shell species groups: Creseidae (*Creseis, Hyalocylis, Styliola*) and Cavoliniidae composed of two sub families, the Cavoliniinae (*Cavolinia Clio and Diacria*) and the Cuvierininae (*Cuvierina*) B) The right topology is deduced from the works of Spoel [13], and Bé & Gilmer [67] which group all the straight shell species in Cavoliniidae, which is composed of three sub-families Clionae (*Clio, Creseis, Hyalocylis, Styliola*), Cuvierininae (*Cuvierina*) and Cavoliniinae (*Cavolinia, Diacria*). Family and sub-family taxa are indicated by symbols: diamond for Limacinidae; down triangle for Cavoliniidae; square for Creseidae; up triangle for Clionae; hexagon for Cavoliniinae; round for Cuvierininae.

Since Meisenheimer [12], the taxonomy of Thecosomata consists of two sub-orders, the Euthecosomata and Pseudothecosomata (Figure 1). The phylogenetic relationships within Pseudothecosomata are well resolved and supported by a consensus between authors who admitted the existence of three families: Peraclidae Cymbuliidae and Desmopteridae. However, the relationships among the Euthecosomata, especially concerning the straight shell species, has been profoundly modified over the last two centuries and can be summarized by two major revisions. The coiled shell species were recognized as the Limacinidae Gray, 1847 and straight shell species as the Cavoliniidae Fisher, 1883. A first modification of the Cavoliniidae has been given by Spoel in 1967 [13] who divided this family in three sub-families: Cuvierininae (*Cuvierina*), Cavoliniinae (*Diacria*, *Cavolinia*) and Clionae (*Creseis*, *Styliola*, *Hyalocylis*, *Clio*) (Figure 1A). This classification was followed until Rampal [14] who proposed a new Thecosomata systematic (Figure 1B). On the basis of the presence of a straight conical shell and morpho-anatomical characteristics, Rampal recognized a new family within the straight shell species, the Creseidae, which consisted of *Creseis*, *Styliola* and *Hyalocylis*. Cavoliniidae were consequently only composed of two sub-families, the Cuvierininae (*Cuvierina*) and the Cavoliniinae (*Diacria* and *Cavolinia-Diacavolinia* complex), which are both characterized by an adult dorso-ventrally depressed peristoma or entire teloconch. According to the same author, the *Clio* genus was moved within the Cavoliniinae, a clade, which is therefore supported by the presence of lateral ridges on the shell. In this last evolutionary scenario the Limacinidae were paraphyletic due to the position of *Thilea helicoides* ( = *Limacina helicoides*), which is the sister group of Cavoliniidae. This paraphyly conflicted with previous authors for which the monophyly of coiled shell described by Gray [15] was never questioned although different infra families levels were debated [13], [16]. In this manuscript, we used the taxonomic nomenclature given by Rampal [17], [14].

Most of the authors accepted that all the existing straight shell species derived from a common ancestor with a coiled shell similar in morphology to *Limacina* or *Peraclis* genera on the basis of ontological [18] and morpho-anatomical data [8], [9], [12], [19], [14], [20]. A widely accepted hypothesis on the straight shell morphogenesis and the developmental re-patterning driving the transition from a coiled to a straight shell was firstly proposed by Boas [8], who pointed the inversion of the trunk organs relative to head organs in the straight shell species and an extension of the visceral sac when compared with the coiled shell species. Boas' hypothesis was later corroborated by the discovery of a coiled shell belonging to the oldest fossils of Euthecosomata ever found (*Spirialis mercinensis* Watelet & Lefèvre, 1885), by incompletely unwinding more recent fossils (e.g. *Camptoceros* Wenz, 1929 and *Bovicornu* Meyer, 1886), and the spiral aragonitic microstructure of the teloconch in straight shell species [17]. Alternatively to the monophyly of straight shell species hypothesis, Rampal [17], [14] proposed the paraphyly of the straight shell species assuming that straight shell innovation is likely homoplasic and occurred independently in two different lineages, on the one hand from a *Limacina*-like ancestor in the Creseidae lineage, and on the other hand from a *Thilea*-like ancestor in the Cavoliniidae lineage (Figure 1B).

More recently, a molecular study based on the mitochondrial gene coding for cytochrome oxydase confirmed the plesiomorphic status of the coiled shell type and suggested the paraphyly of the Limacinidae [21], hypothesized by Rampal [14] ( see Figure 1B). Thus, the debate about the evolution of the shell and the conclusions of Jennings and Coll. reveal the need of new molecular data in Thecosomata to infer phylogenetic hypotheses based on traditional characters. However, molecular studies are limited when the sampling in a given clade is not representative of the current biodiversity. This problem is exacerbated in zooplankton groups such as Thecosomata that spread all over the world's oceans at all bathymetric levels. Recent around-the-world expeditions such as *Tara* Oceans collected plankton with complete environmental parameters at different depths [22]. In this study, the broad sampling of *Tara* Oceans from 153 stations worldwide over the past three years and a various set of others regional missions allowed us to highlight the molecular phylogeny of Thecosomata with a new set of DNA sequences. We assessed the phylogenetic analysis of two genes, the large subunit of 28S rRNA (28S) and the cytochrome oxidase subunit 1 (COI). We also revised the morpho-anatomical taxonomy of Thecosomata by performing the first cladistic analysis based on 55 character comprehensive taxonomies. Finally, coupled with the current paleontological knowledge, we proposed a new evolutionary scenario for Euthecosomata with the revival of Orthoconcha.

## Materials and Methods

### Data collections for the morphological analysis

Morphological matrix was developed from a collection of specimens originated from ancient expeditions. The morphological identification was made according to the determination key published by Rampal [23]. The Formol fixation and the long stay in Ethanol of these specimens did not allow us to use them for the molecular analysis. These sampling were carried out during oceanic expeditions performed on the following ships: Thor (1910), Dana (1921, 1930), Président-Théodore-Tissier (1957–1958), Shoyo-Maru (1959), Thalassa (1961, 1963, 1969, 1977), Argonaut (1965), Jean-Charcot (1966, 1979, 1981), Ariadne (1966), Magga Dan (1966–1967), Coriolis (1967–1969), Korotneff (1970–1971), La Coquille (1971), Marion-Dufresne (1981, 1982, 1986). Morphological identifications of specimens were performed using a stereoscopic microscope Wild M5 and photonic microscope Wild M10. Moreover, a scanning electron microscope Philips XL30ESEM was used to study both the micro-architectural structure of shell and radular teeth morphology.

### Molecular analysis: data collections, DNA extraction, amplification and sequencing

In-group sampling included specimens collected from various stations of Tara expedition and also from other regional missions (see Table 1) which were performed on overall Oceans: North-South Atlantic Ocean, Gulf of Mexico, Pacific Ocean, Mediterranean Sea, Adriatic Sea, Red Sea, North-South Indian Ocean, Persian Gulf and Mozambique Channel. Genomic DNA was extracted from overall body of each specimen using the DNAeasy kit (Qiagen, Valencia, CA). The morphological identification of specimens was made according to the determination key [23]. A 660 bp fragment of the COI gene was amplified using the primers LCO-1490 (5′-GGTCAACAAATCATAAAGATATTGG-3′) and HCO-2198 (5′-AAACTTCAGGGTGACCAAAAAATCA-3′) previously designed by Folmer et al. [24]. A fragment of rRNA 28S gene (approximately about 1000 pb) was amplified using the following primers from Dayrat et al. [25] 28SC1 (5′-ACCCGCTGAATTTAAGCAT-3′) and 28SD3 (5′-GACGATCGATTTGCACGTCA-3′). PCRs were performed in 50 μL volumes with the following reagents: 1X PCR buffer (Taq PCR core kit, Qiagen), 0.2 mM of each dNTPs mix, 0,5 mM of each primer, 2 to 4 ul (depending on DNA concentration) of extracted genomic DNA, and 1U of Taq polymerase. Reactions were cycled under the following protocol: initial denaturation, 95°C for 5 min.; 40 cycles of 95°C for 30 sec., 55°C for 45 sec, 72°C for 1 min; final extension, 72°C for 5 min. Purification and sequencing of PCR products were then performed using ABI BigDye Terminators v3.1 and electrophoresed on an ABI 3730 Automated DNA Sequencer by GATC Biotech (Konstanz, Germany). All fragments were sequenced in both directions using the amplicon primers. Both sequences of the same specimen were compiled using SeqScape version 2.5.

**Table 1:** Origins of the specimens of the molecular analysis.

# id	Species name	COI	28S rRNA	Mission	Origin	Genbank ID
163	***Cavolinia flava***	+	+	ECOSUR	Caribean Sea (Yucatan/Belize)	KC774033/KC774104
216	***Cavolinia globulosa***		+	TARA St 41	Gulf of Aden	KC774101
224	***Cavolinia globulosa***		+	TARA St 42	N. Ind. Oc. (Maldives)	KC774141
276	***Cavolinia labiata***	+		TARA St 52	Ind. Oc. (East of Madagascar)	KC774038
265	***Cavolinia labiata***	+	+	TARA St 66	S. Atl. Oc. (Cape Town)	KC774037/KC774099
263	***Cavolinia inflexa***	+		TARA St 66	S. Atl. Oc. (Cape Town)	KC774036
164	***Cavolinia inflexa***	+		ECOSUR	Caribean Sea (Yucatan/Belize)	KC774031
262	***Cavolinia inflexa***	+	+	TARA St 66	S. Atl. Oc. (Cape Town)	KC774030/KC774102
114	***Cavolinia inflexa***		+	ANTEDON	Gulf of Lyon (Cassidaigne) Med. Sea	KC774103
237	***Diacavolinia longirostris***	+	+	ECOSUR	Caribean Sea (Yucatan/Belize)	KC774055/KC774121
255	***Diacavolinia longirostris***	+		TARA St 58	Mozambique Channel	KC774035
311	***Diacavolinia longirostris***		+	CRER 2	Caribean Sea (Virgin Islands)	KC774122
310	***Diacavolinia longirostris***		+	CRER 2	Caribean Sea (Virgin Islands)	KC774123
313	***Diacavolinia longirostris***		+	CRER 2	Caribean Sea (Virgin Islands)	KC774119
235	***Diacavolinia longirostris***		+	ECOSUR	Caribean Sea (Yucatan/Belize)	KC774120
236	***Diacavolinia longirostris***		+	ECOSUR	Caribean Sea (Yucatan/Belize)	KC774124
128	***Diacavolinia longirostris***		+	ECOSUR	Caribean Sea (Yucatan/Belize)	KC774118
284	***Cavolinia sp***	+	+	TARA St 51	Ind. Oc. (East of Madagascar)	KC774034/KC774100
215	***Cavolinia sp***	+		TARA St 42	N. Ind. Oc. (Maldives)	KC774032
84	***Clio pyramidata***	+		CRER 2	Caribean Sea (Yucatan/Belize)	KC774067
118	***Clio pyramidata***	+	+	ANTEDON	Gulf of Lyon (Cassidaigne) Med. Sea	KC774065/KC774096
193	***Clio pyramidata***	+		CRER 2	Caribean Sea (Virgin Islands)	KC788279
317	***Clio pyramidata***	+	+	CRER 2	Caribean Sea (Yucatan/Belize)	KC774068/KC774095
231	***Clio pyramidata***	+	+	TARA St 52	Ind. Oc. (East of Madagascar)	KC774066/KC774097
282	***Clio convexa***	+	+	TARA St 53	Ind. Oc. (East of Madagascar)	KC774069/KC774093
206	***Clio convexa***	+		TARA St 34	Red Sea	KC774062
223	***Clio convexa***	+	+	TARA St 42	N. Ind. Oc. (Maldives)	KC774063/KC774105
291	***Clio convexa***		+	TARA St 34	Red sea	KC774094
292	***Clio convexa***		+	TARA St 34	Red sea	KC774092
213	***Clio cuspidata***	+	+	TARA St 42	N. Ind. Oc. (Maldives)	KC774064/KC774098
166	***Diacria trispinosa***	+	+	ECOSUR	Caribean Sea (Yucatan/Belize)	KC774073/KC774117
264	***Diacria trispinosa***	+		TARA St 66	S. Atl. Oc. (Cape Town)	KC774078
165	***Diacria major***	+	+	ECOSUR	Caribean Sea (Yucatan/Belize)	KC774072/KC774115
256	***Diacria quadridentata***	+	+	TARA St 58	Mozambique Channel	KC774077/KC774114
325	***Diacria quadridentata***	+		TARA St 76	SW. Atl. Oc.	KC774080
326	***Diacria quadridentata***	+		TARA St 76	SW. Atl. Oc.	KC774081
198	***Diacria quadridentata***	+		CRER 2	Caribean Sea (Yucatan/Belize)	KC774074
200	***Diacria quadridentata dana***	+	+	CRER 2	Caribean Sea (Yucatan/Belize)	KC774075/KC774113
280	***Diacria quadridentata dana***	+		TARA St 50	N. Ind. Oc.	KC774076
160	***Diacria rampali***		+	ECOSUR	Caribean Sea (Yucatan/Belize)	KC774116
95	***Hyalocylis striata***	+	+	FED IRD	Pac. Oc (French Polynesia)	KC774061/KC774146
222	***Hyalocylis striata***	+	+	TARA St 18	Mediterranean Sea	KC774059/KC774144
190	***Hyalocylis striata***	+	+	CRER 2	Caribean Sea (Yucatan/Belize)	KC774057/KC774143
233	***Hyalocylis striata***	+	+	TARA St 34	Red sea	KC774060/KC774147
185	***Hyalocylis striata***	+	+	TARA St 14	Mediterranean Sea	KC774056/KC774142
191	***Hyalocylis striata***	+	+	CRER 2	Caribean Sea (Yucatan/Belize)	KC774058/KC774145
119	***Peraclis reticulata***	+	+	ANTEDON	Gulf of Lyon (Cassidaigne) Med. Sea	KC774089/KC774160
196	***Peraclis reticulata***	+	+	CRER 2	Caribean Sea (Yucatan/Belize)	KC774088/KC774162
270	***Peraclis reticulata***		+	TARA St 32	Red Sea	KC774164
307	***Peraclis reticulata***		+	CRER 2	Caribean Sea (Yucatan/Belize)	KC774165
194	***Peraclis reticulata***		+	CRER 2	Caribean Sea (Yucatan/Belize)	KC774161
212	***Peraclis reticulata***		+	TARA St 42	N. Ind. Oc. (Maldives)	KC774163
234	***Cymbulia sp***	+	+	TARA St 30	E. Med. Sea	KC774090/KC774159
172	***Cymbulia sp***		+	ECOSUR	Caribean Sea (Yucatan/Belize)	KC774158
173	***Styliola subula***		+	ECOSUR	Caribean Sea (Yucatan/Belize)	KC774110
121	***Styliola subula***		+	ANTEDON	Gulf of Lyon (Cassidaigne) Med. Sea	KC774112
229	***Styliola subula***		+	TARA St 52	Ind. Oc. (East of Madagascar)	KC774111
189	***Styliola subula***		+	CRER 2	Caribean Sea (Yucatan/Belize)	KC774109
277	***Cuvierina urceolaris***	+	+	TARA St 52	Ind. Oc. (East of Madagascar)	KC774071/KC774107
85	***Cuvierina columnella***	+		CRER 2	Caribean Sea (Yucatan/Belize)	KC774070
304	***Cuvierina columnella***		+	TARA St 98	S.E. Pac. Oc.	KC774106
324	***Cuvierina spoeli***		+	TARA St 64	Mozambique Channel	KC774108
219	***Creseis chierchae***	+	+	TARA St 41	Gulf of Aden	KC774044/KC774137
109	***Creseis chierchae***		+	CRER 2	Caribean Sea (Yucatan/Belize)	KC774136
75	***Creseis chierchae***		+	CRER 2	Caribean Sea (Yucatan/Belize)	KC774138
210	***Creseis chierchae***	+		TARA St 42	N. Ind. Oc. (Maldives)	KC774043
272	***Creseis acicula***	+	+	TARA St 52	Ind. Oc. (East of Madagascar)	KC774051/KC774127
126	***Creseis acicula***	+	+	ANTEDON	Gulf of Lyon (Cassidaigne) Med. Sea	KC774054/KC774126
82	***Creseis acicula***	+	+	CRER 2	Caribean Sea (Yucatan/Belize)	KC774053/KC774125
218	***Creseis acicula***	+	+	TARA St 41	Gulf of Aden	KC774052/KC774134
124	***Creseis acicula***		+	ANTEDON	Gulf of Lyon (Cassidaigne) Med. Sea	KC774135
81	***Creseis conica***		+	CRER 2	Caribean Sea (Yucatan/Belize)	KC774140
115	***Creseis conica***	+		ANTEDON	Gulf of Lyon (Cassidaigne) Med. Sea	KC774039
221	***Creseis conica***	+	+	TARA St 18	Mediterranean Sea	KC774042/KC774139
159	***Creseis conica***	+		ECOSUR	Caribean Sea (Yucatan/Belize)	KC774041
125	***Creseis conica***	+		ANTEDON	Gulf of Lyon (Cassidaigne) Med. Sea	KC774040
157	***Creseis virgula***	+	+	ECOSUR	Caribean Sea (Yucatan/Belize)	KC774045/KC774128
271	***Creseis virgula***	+	+	TARA St 32	Red Sea	KC774050/KC774133
250	***Creseis virgula***	+	+	TARA St 34	Red Sea	KC774048/KC774131
207	***Creseis virgula***	+	+	TARA St 34	Red Sea	KC774046/KC774129
269	***Creseis virgula***	+	+	TARA St 34	Red Sea	KC774049/KC774132
214	***Creseis virgula***	+	+	TARA St 42	N. Ind. Oc. (Maldives)	KC774047/KC774130
78	***Limacina inflata***	+		CRER 2	Caribean Sea (Virgin Islands)	KC774086
111	***Limacina inflata***	+		ANTEDON	Gulf of Lyon (Cassidaigne) Med. Sea	KC774079
170	***Limacina inflata***	+		ECOSUR	Caribean Sea (Yucatan/Belize)	KC774082
99	***Limacina inflata***		+			KC776157
228	***Limacina inflata***	+		TARA St 23	Mediterranean Sea	KC774085
285	***Limacina helicina***	+		ECOSUR	Caribean Sea (Yucatan/Belize)	KC774083
286	***Limacina helicina***	+	+	TARA St 85	Ant. Oc.	KC774084/KC774156
329	***Limacina helicina***	+	+	TARA St 66	S. Atl. Oc. (Cape Town)	KC774087/KC774155
278	***Limacina lesueurii***		+	TARA St 52	Ind. Oc. (East of Madagascar)	KC774154
305	***Limacina trochiformis***		+	CRER 2	Caribean Sea (Virgin Islands)	KC774153
268	***Limacina bulimoides***		+	TARA St 66	S. Atl. Oc. (Cape Town)	KC774148
197	***Limacina bulimoides***		+	CRER 2	Caribean Sea (Virgin Islands)	KC774152
308	***Limacina bulimoides***		+	CRER 2	Caribean Sea (Virgin Islands)	KC774151
174	***Limacina bulimoides***		+	ECOSUR	Caribean Sea (Yucatan/Belize)	KC774150
309	***Limacina bulimoides***		+	CRER 2	Caribean Sea (Virgin Islands)	KC774149
281	***Desmopterus sp***		+	TARA St 53	Mozambique Channel	KC774166
225	***Desmopterus sp***		+	TARA St 40	Gulf of Aden	KC774167
105	***Gymnosome sp***	+	+	FED IRD	Pac. Oc (French Polynesia)	KC774091/KC774168

The samples and mission correspondence are indicated as Ind. Oc.: Indian Ocean. N. Ind. Oc: North Indian Ocean. S. Atl. Oc.: South Atlantic Ocean. E. SW. Atl. Oc.: South West Atlantic ocean. S. E. Pac. Oc.: South East Pacific Ocean. Ant. Oc.: Antarctic Ocean. Med.: East Mediterranean Sea. St Number corresponds to the TARA station reference.

We completed this sequence data set by sequences available in public databases used for this study (see Table 2).

**Table 2:** Supplementary molecular data from genbank and the Bar Coding of Life Database.

Species name	COI	28SrRNA	ID
***Creseis acicula***		+	gi|82502297|gb|DQ237982.1|
***Cuvierina columnella***		+	gi|82502299|gb|DQ237984.1|
***Cuvierina columnella***	+		gi|82502319|gb|DQ237998.1|
***Diacria quadridentata***		+	gi|82502302|gb|DQ237987.1|
***Diacria quadridentata***	+		gi|82502325|gb|DQ238001.1|
***Clio pyramidata***		+	gi|82502301|gb|DQ237986.1|
***Clio pyramidata***	+		gi|82502323|gb|DQ238000.1|
***Cavolinia uncinata***		+	gi|82502298|gb|DQ237983.1|
***Cavolinia uncinata***	+		gi|82502317|gb|DQ237997.1|
***Cavolinia inflexa***	+		BOLD AAM3343
***Hyalocylis striata***	+		gi|82502321|gb|DQ237999.1|
***Peraclis valdiviae***	+		gi|241947896|gb|FJ876940.1|
***Peraclis bispinosa***	+		gi|241947890|gb|FJ876937.1|
***Peraclis bispinosa***	+		gi|241947894|gb|FJ876939.1|
***Cymbulia sibogae***	+		gi|241947880|gb|FJ876932.1|
***Corolla spectabilis***	+		gi|241947886|gb|FJ876935.1|
***Gleba cordata***	+		gi|241947882|gb|FJ876933.1|
***Gleba cordata***	+		gi|241947884|gb|FJ876934.1|
***Clione limacina***	+		gi|284504735|gb|GU227107.1|
***Spongiobranchaea australis***		+	gi|82502303|gb|DQ237988.1|
***Spongiobranchaea australis***	+		gi|82502327|gb|DQ238002.1|
***Pneumoderma atlantica***	+		gi|82502329|gb|DQ238003.1|
***Limacina helicina helicina***	+		gi|37933603|gb|AY227379,1|
***Limacina helicina antartica***	+		gi|270310122|gb|GQ861831.1|
***Limacina helicina***	+		gi|270310124|gb|GQ861832.1|
***Limacina trochiformis***		+	gi|82502294|gb|DQ237979.1|
***Thilea helicoides***	+		gi|241947866|gb|FJ876925.1|

### Morphological analysis

Morphological data were analysed using Paup* 4.0b10 [26] under maximum parsimony (MP) with a heuristic search with 10 random taxon addition replicates followed by tree bisection and reconnection (TBR) branch swapping. All characters were treated as unordered and un-weighted. The Deltran optimization was used to map the character changes on tree. To evaluate how homoplasy impacts the optimal topology, we use the g1 statistic on 10^6^ randomly sample trees for the compete data set (56 taxa) and for the partial data set (without the species with identical « sequence », 28 taxa). Clade frequencies were obtained by 50% majority-rule consensus trees. Clade supports were assessed by bootstrapping (500 with 20 random addition replicates each). Due to the absence of homologous characters between Gymnosomata and Thecosomata (for the selected characters) it was not possible to use a Gymnosomata as out-group. Thus we choose the *Desmopterus* species (Pseudothecosomata) as out-group.

### Molecular analysis

#### Sequence alignments

For the coding COI gene, the sequences were firstly aligned in protein and then converted in nucleotide using ClustalW implemented in the software package MEGA Version 5 Beta [27]. This method allowed us to maximise homology between nucleotidic positions when amino acid deletion/insertion occurred.

Considering the 28S gene, the alignment of nucleotidic sequences was done using ClustalW implemented in the software package MEGA Version 5 Beta and refined by eye using the secondary structure information. Nucleotidic ambiguities usually occur in the loop region. We use the programme Aliscore [28], [29] to test the impact of high heterogeneity site that could affect in a negative way the phylogenetic reconstruction and therefore be considered as a noisy” sites. We use the “-N” and “-N –r –w4” parameter for both molecular markers (COI and 28S).

#### Phylogenetic single-gene analyses and model selection

The goal of partitioning is to divide the sequences into regions that have evolved under different evolutionary models. The more partitions, the more accurately the data is modelled. However, as the number of nucleotide positions per partition decreases, the amount of random errors associated with estimating parameters for each partition increases. Considering these different parameters, each partition was conducted using the Maximum Likelihood (ML) using the MEGA Version 5. A random starting tree was generated using the Neighbour-Joining method with the partial deletion option selected (75% site coverage cut-off). The best DNA model was selected using BIC (Bayesian information criterion). Therefore, we attempted to achieve a balance between partitioning the data into similar units and over partitioning. Partitions were chosen *a priori* based on gene identity (i.e. COI and 28S) and general biochemical or evolutionary constraints (i.e. codon positions, stems and loops). Appropriate evolutionary models were chosen for each partition using the likelihood ratio test (LRT).

A Maximum Likelihood tree was estimated using the Nearest-Neighbour-interchange (NNI) option under the best partition strategy. Topological robustness was investigated using 1000 non-parametric bootstrap replicates. Branches with bootstrap values higher than 70% were considered well supported [30].

We also performed Bayesian phylogenetic analyses using MrBayes 3.0b4 [31]. Each analysis consisted of 2.10^7^ generations with a random starting tree, default priors, the same set of branch lengths for each partition, and four Markov chains (with default heating values) sampled every 1000 generations. Adequate burn-in was determined by examining a plot of the likelihood scores of the heated chains for convergence on stationarity as well as the effective sample size (ESS) of values in Tracer 1.5 [32].

To test the impact of “noisy sites” we compute maximum likelihood phylogenetic analyses using PHYML-aBayes 3.0.1 beta programme [33] [34] on the complete data set (with “noisy” sites) and the partial data set (without “noisy” sites) for COI, 28S and 28S+COI. We calculate two non-parametric branch support (Bootstrap and SH-aLRT) and two parametric branch support (aBAYES and aLRT) as developed in [35], [34]. We use bootstrap and aBAYES values to establish a criterion of “quality”. When bootstrap value was “low” but the other three were “high” then we considered a potential false negative support; if bootstrap value was “high” but the other three were “low” then we consider a potential false positive support.

#### Time divergence estimation: Pairwise genetic distances based method and Relaxed Bayesian molecular clock

To estimate time divergence, it is generally assumed that sequences evolve following a roughly constant rate over time (i.e. the molecular clock hypothesis). However, this evolutionary rate is dependent on many factors including the underlying mutation rate, metabolic rate, generation times, population sizes and selective pressure [36]. All these parameters are extremely difficult to estimate and an abusive use may induce a violation of the strict molecular clock hypothesis. However, it is possible to perform statistical tests that evaluate how the evolutionary rates along the branches in a given tree deviate from a constant rate. This way, the uncorrelated relaxed molecular clock method could be used on a non-fixed tree topology and the parameter estimated by averaging over a set of plausible trees using MCMC [37].

In order to have the most integrative estimation of divergence time we used the similar approaches than in Fouquet et al. [38] by combining two different methods. This two methods were performed both on the concatenate sequences data set with noisy site and on the concatenated sequences data set without “noisy” sites.

The first method for estimating the diversifying timing is based on the analysis of the distribution of pairwise genetic distance within Thecosomata. It was implemented in the R language [39]. One considers here that the pairwise distances distribution among sequences reflects the timing of evolutionary [40]. By example, a sudden diversifying event could generate a high number of lineages of similar age. In such a case, the distribution of pairwise distances is expected to exhibit modes corresponding to the origin of different lineages and to differences among closely related haplotypes within each lineage. In contrast, a more continuous process of diversifying would generate a smoother distribution. Such interpretation is valid under the assumption of molecular clock. So, sequences which depart significantly from the molecular clock hypothesis were removed. To do this, the branch length test was performed. To achieve this goal, we used a neighbour-joining tree based on concatenated data set (40 sequences) using the selected substitution model (Table S3). Then, we examined the deviation of the root-to-tip distance from the average for all sequences excepted for the out-group sequences (see [41], p199 for more details).

From the remaining sequences, we estimated the pairwise distribution considering a kernel estimate based on the Gaussian density. Using the density function of R with default values, allowed us to define 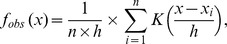 with 
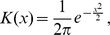
 in which h = smoothing parameter, n = number of observations, x_i_ = observation (pairwise distance). Discrepancy between (i) the estimated pairwise distances distribution “fobs” and (ii) the expected pairwise distribution under a null model (H0) of diversification was then tested. Let define (H0): “The process responsible for the observed distribution is a simple birth–death process with constant rates across time” versus (H1): “The process responsible for the observed distribution departs from a simple birth–death process”. Let us define p_H0_ as the theoretical pairwise distribution under (H_0_). This distribution p_H0_ was estimated from simulations of pure birth-death process, where birth and death rate parameters were previously estimated from the maximum likelihood estimators proposed by [42]. To test (H_0_) against (H_1_), we considered the same statistic and procedure as proposed by Fouquet et al. : see [38] for more details. When the discrepancy between the observed and theoretical distribution is significant, an examination of their respective distributions may help for identifying the diversifying rates corresponding to local maxima of the distribution. In this way, we identified all the different maxima of f_obs_(x) considering the sign of [f_obs_(x)-f_obs_(x-Δ)* f_obs_(x)-f_obs_(x+Δ)], Δ being the discretization step of x-axis. The major advantage of this method is that the distribution and timing of divergence events are inferred without relying on any phylogenetic priors among species.

The second analysis was based on a relaxed Bayesian molecular clock with uncorrelated lognormal rates (Beast 1.4.6, [32]). Then a concatenated data set composed of two partitions (corresponding to the 28S and COI part) was done considering the best model for each partition. We used an estimated time of 56.5 Ma for the basal split of Euthecosomata (normal distribution, standard deviation  = 1) that constitutes a corner stone of the paleontological knowledge [43], [44], [45], [46]. Furthermore, this time divergence is very closed to the following split occurring between Limacinidae and other Euthecosomata families (about 54.0 to 52.0 Ma). To define group priors, we considered two different approaches: first by not defining any monophyletic group (whatever the taxonomic rank) and second by selecting only three Orders, Gymnosomata, Pseudothecosomata and Euthecosomata, as monophyletic groups.

The tree prior used the Yule Process of speciation, with a randomly generated starting tree. The operators were optimized by a preliminary run of 10^6^ generations sampled every 1000 generations followed by two independent runs of 5.10^7^ generations sampled every 5.10^4^ generations. Adequate burn-in was determined by examining a plot of the likelihood scores of the heated chain for convergence on stationarity. We used the overall estimates of the rates of molecular evolution based on the concatenated COI and 28S data set.

## Results

### Morphological data

Among the 55 characters, 47 were informative for the cladistic analysis. The Heuristic MP search found more than 100,000 trees 103 steps long and displayed a g1 equal to −0.46. The consistency index was equal to 0.816 (RI  = 0.854) and the Homoplasy index equal to 0.185.When we remove species with identical coding morphological sequence (28 taxa, 50% of the data set) we found 74,840 trees and a g1 equal to −0.65. This result indicated that identical sequences induce homoplasy. The consistency index was equal to 0.816 (RI  = 0.854) and the Homoplasy index equal to 0.185.

**Figure 2:**
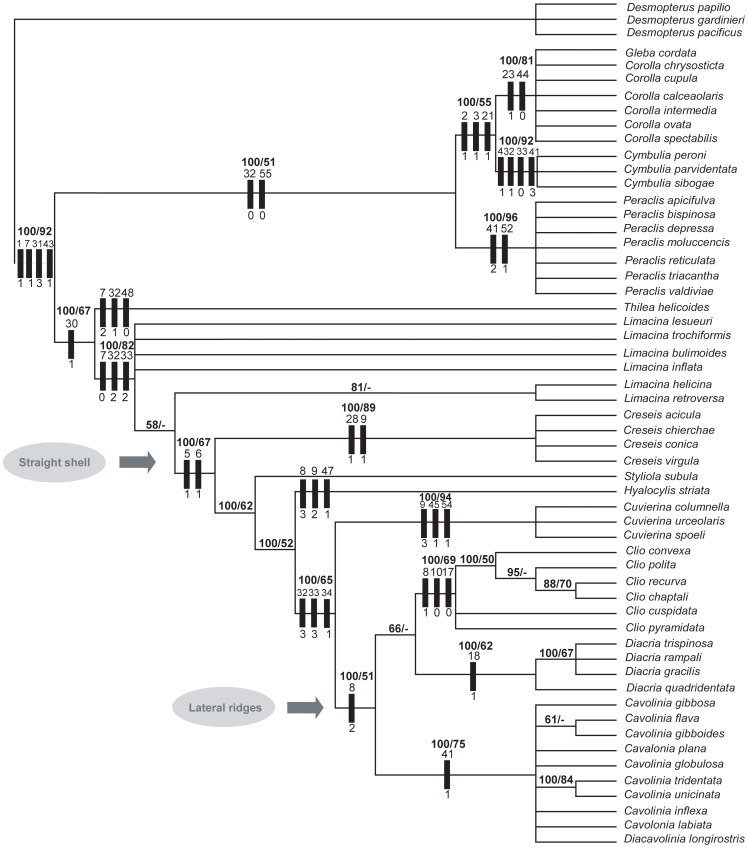
Cladistical analysis of morphological data. Majority rule consensus tree of 74,840 equally parsimonious tree (CI = 0.816; RI = 0.854). Majority rule consensus values and bootsrap values are respectively shown above internal branches (only values ≥50% are shown). Only synapomorphies presenting a consistency index  = 1 are shown on the branches. Black bars represent the synapomorphy characterized by the corresponding morphological character number and the character state change respectively above and below. Characters coding is presented in Table S1.

Rooted on a Pseudothecosomata belonging to *Desmopterus*, the majority-rule consensus tree (first value) and the bootstrapped tree (second value) displayed identical topologies (Figure 2). The other Pseudothecosomata (represented by four genera and 17 species) constituted a monophyletic group (100/51) supported by two synapomorphies (characters #32 pallial gland with one zone of parallepipedic cells and #55 three visceral ganglia). Furthermore, when Pseudothecosomata were considered as a monophyletic group (*Peraclis*, *Cymbulia*, *Corolla*, *Gleba* and *Desmopterus*) the evolutionary scenario observed for Euthecosomata was identical. The monophyly of the Euthecosomata was also well supported (100/67), although only one character was homologous to Pseudothecosomata (#30 rhinophora).

Considering Euthecosomata families, the first cladogenesis event separated *Thilea helicoides* from the other Euthecosomata species inducing the paraphyly of Limacinidae. In addition, *Limacina* genus itself was represented by two paraphyletic subgroups, a first (*L. lesueurii*, *L. trochiformis*, *L. bulimoides*, *L. inflata*) with a basal polytomy and a second (*L. helicina* and *L. retroversa*) supported by weak support values (81/−). Furthermore, the Creseidae family (*Creseis*, *Styliola* and *Hyalocylis*) was a paraphyletic assemblage. The only monophyletic Euthecosomata family was the Cavoliniidae (100/65), characterised by three synapomorphies (#32 pallial gland with three zones of differentiated cells, #33 pallial gland with three zones of parallelepipedic cells and #34 pallial gland with simple and crateriform cells). Also the Cavoliniinae sub-family represented by *Clio*, *Diacria* and *Cavolinia-Diacavolinia* complex constituted a monophyletic group (100/51) characterized by straight and dorso-ventraly depressed shell with lateral ridges (#10).

Interestingly, the morphological analysis detected a new clade containing the Creseidae and Cavoliniidae, which exhibited two synapomorphic characters (#5 symmetrical straight shell and #6 helicoidal organisation of the aragonitic micro-architecture of the shell).

Finally, except for *Limacina* and both monospecific genus *Styliola* and *Hyalocylis,* all Euthecosomata genera appeared to be monophyletic and natural groups. They are *Creseis* (100/89), *Cuvierina* (100/94,) *Cavolinia-Diacavolinia* (100/75), *Clio* (100/69) and *Diacria* (100/62).

### Molecular data

#### Cytochrome oxydase sub-unit I

The sequencing of 63 samples succeeded (Table 1), constituting with genbank sequences (Table 2) a total data set of 83 sequences. The complete alignment displayed 657 positions for which 440 were variable including 428 parsimony-informative sites. The selected model for the first position was TN93 +Γ+ I (lnL  = −3333.798), for the second position GTR + Γ (lnL  = −1299.116) and for the third HKY + Γ (lnL  = −9216.247). The sum of lnL was equal to −13849.162 (see Table S3). The selected model for the whole data set was GTR + Γ+ I with a lnL equal to −15018.725. The LRT was highly significant between the two lnL, so we used the models that estimated the evolution for each codon position. Considering the partial data set elaborated without “noisy sites”, 50 sites (7.61%) are filtering out. The selected model for the data set without “noisy sites” was GTR + Γ+ I with a lnL equal to −13320.299.

**Figure 3:**
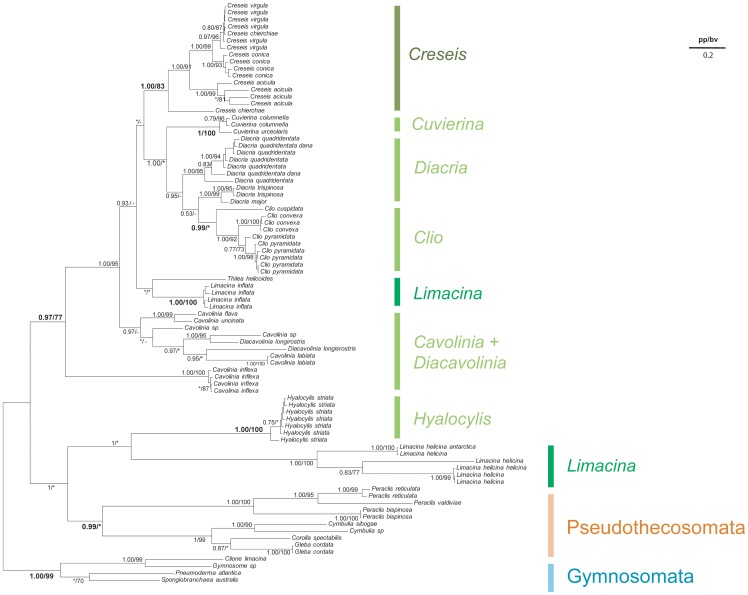
Phylogenetic analysis of Thecosomata based on COI data. We display the topology of the Bayesian tree issued from the COI gene complete data set with “noisy” site (657 base pair) and for each clade, the posterior probability (pp) is indicated, followed by the maximum likelihood bootstrap values (bv). Non supported group (pp<0.5; bv<70%) are indicated by stars. Topological incongruences between Bayesian tree and Maximum likelihood tree are indicated by hyphens. Evolutionary rate is indicated by scale bar.

Rooted on Gymnosomata, Euthecosomata constituted a paraphyletic group (Figure 3) because *Hyalocylis striata* and *Limacina helicina* were the sister groups to Pseudothecosomata (1.00/60). Such a result induced the breakdown of Limacinidae which consisted of two distinct groups, first *Limacina helicina* (1.00/100) and second *Thilea helicoides* + *Limacina inflata* (1.00/100). However, contrarily to the paraphyly observed in the morphological analysis, the Limacinidae appeared here polyphyletic.

Cavoliniidae recognized as *Clio*, *Diacria, Cavolinia-Diacavolinia* and *Cuvierina* were polyphyletic from the Bayesian analysis and paraphyletic from the ML analysis while Creseidae represented here by *Creseis* and *Hyalocylis* were polyphyletic in both methods.

At the generic level, only *Creseis*, *Cuvierina*, and *Clio* constituted well-supported monophyletic groups (respectively 1.00/83, 1.00/100, 1.00/65). *Cavolinia inflexa* was the sister group of the other Euthecosomata (0.97/77), inducing the paraphyly of the *Cavolinia* genus. The other *Cavolinia* species *(Cavolinia*-*Diacavolinia*) grouped together (0.97/56) as the sister group to the second *Limacina* group (*L. inflata* + *Thilea helicoides*) plus four other genera (*Clio*, *Diacria*, *Cuvierina*, and *Creseis*) (1.00/95). *Diacria* were paraphyletic with respect to *Clio* from the Bayesian analysis but were monophyletic from the ML analysis, although weakly supported (60).

**Figure 4:**
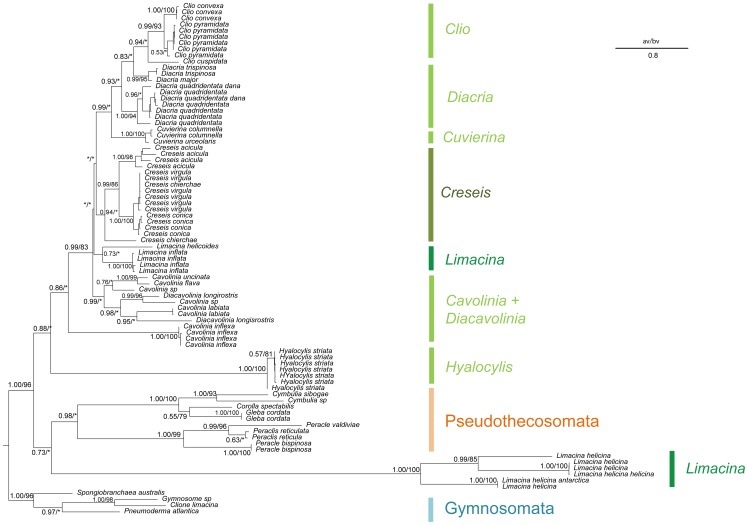
Phylogenetic analysis of Thecosomata based on partial COI data set. We display the topology of the Bayesian tree issued from the COI gene data set without “noisy” site ( 607 base pair). For each clade, the maximum likelihood bootstrap values (bv) and the a-Bayes value (av) are indicated. Non supported group (av<0.5; bv<70%) are indicated by stars. Topological incongruences between a-Bayes tree and bootstrap tree are indicated by hyphens. Evolutionary rate is indicated by scale bar.

Considering the partial data set (without “noisy” sites, 607 pb), the most striking result was the position of *Hyalocylis striata* (Figure 4). Indeed, this species do not constituted the sister group to *Limacina helicina* but the sister group of the other Euthecosomata. Bootstrap value was “low” however the aBayes value was equal to 0.88 (0.97 for the aLRT, data not shown) suggesting that this phylogenetic position was underestimated.

#### Large subunit 28S Rrna

The sequencing of 77 samples succeeded (Table 1), constituting with genbank sequences (Table 2) a total data set of 84 sequences. The complete alignment displayed 1013 positions for which 557 were variables including 432 parsimony-informative sites. The selected model for stems was K2P + Γ (lnL  = −2353.853) and for loops GTR + Γ (lnL  = −5442.662). The sum of lnL was equal to −7796.515 (see Table S3). The selected model for the whole data set was GTR + Γ and presented an lnL equal to −7610.543. The LRT was highly significant between the two lnL, so we used the model that estimated the evolution for the whole data set. Considering the partial data set elaborated without “noisy sites”, 125 sites (12.34%) are filtering out. The selected model for the data set without “noisy sites” was GTR + Γ with a lnL equal to −5730.399.

**Figure 5:**
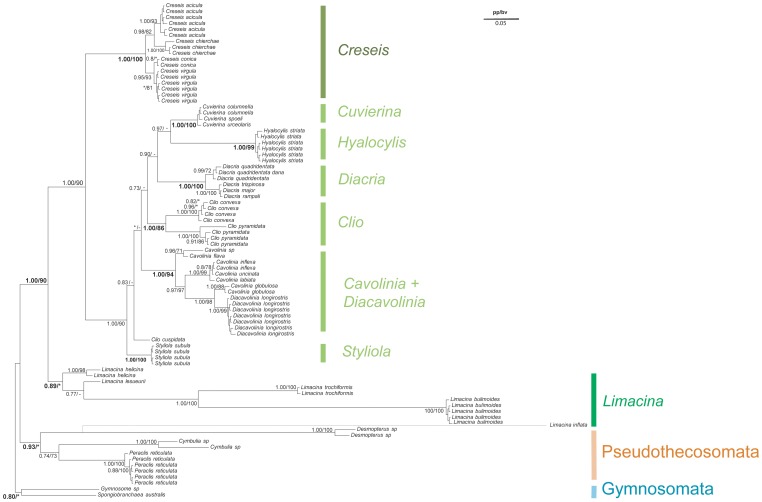
Phylogenetic analysis of Thecosomata based on 28S molecular data. We display the topology of the Bayesian tree issued from the 28S gene complete data set with “noisy” sites (1013 base pair). For each clade, the posterior probability (pp) is indicated, followed by the maximum likelihood bootstrap values (bv). Non supported group (pp<0.5; bv<70%) are indicated by stars. The dotted line corresponds to the topological position of *L.inflata* obtained when the corresponding sequence is added into the data set. Topological incongruences between Bayesian tree and Maximum likelihood tree are indicated by hyphens.

As for the phylogenetic reconstruction based on COI sequences, the 28S tree was rooted on Gymnosomata (Figure 5). One of the most striking result is the position (and the branch length) of *Limacina inflata*. Indeed, it is clear that this sequence evolves faster than the rest of the species studied in this phylogeny. The sequence presented a variable alignment nested between two conserved regions. We obtained this sequence three times using three independent PCR. Moreover this sequence was the sister group of the Thecosomata+ Gymnosomata group using phylogenetic analysis and blast result indicated a higher similarity with this group than other metazoan groups. This result suggests a possible pseudogene but not a contamination. The Pseudothecosomata, represented here by the genera *Peraclis*, *Cymbulia*, and *Desmopterus* were monophyletic with high support from Bayesian analysis only (0.93/57). The Pseudothecosomata were the sister group to all species belonging to Euthecosomata, the monophyly of which was unambiguously found in both methods (1.00/90). In contrast to the results obtained with COI sequences, 28S tree supported most of the Euthecosomata clades traditionally established at infra-generic level but several traditional families were not monophyletic. The Creseidae were polyphyletic from the Bayesian analysis but paraphyletic from the ML analysis because of the variable position of *Hyalocylis striata*. Also the Cavoliniidae were paraphyletic because *Hyalocylis* constituted the sister group to *Cuvierina* from the Bayesian analysis (0.97) although their monophyly was weakly supported in the ML analysis (48). Interestingly, while COI tree did not support the monophyly of *Limacina*, the four species represented here (*L. lesueurii*, *L. helicina*, *L. bulimoides*, *L. trochiformis*) grouped together in a same clade at the basis of all Euthecosomata. Yet *Limacina* genus (set apart *L. inflata*) received marginal support values from Bayesian analysis and low support values from the ML analysis (0.89/62).

**Figure 6:**
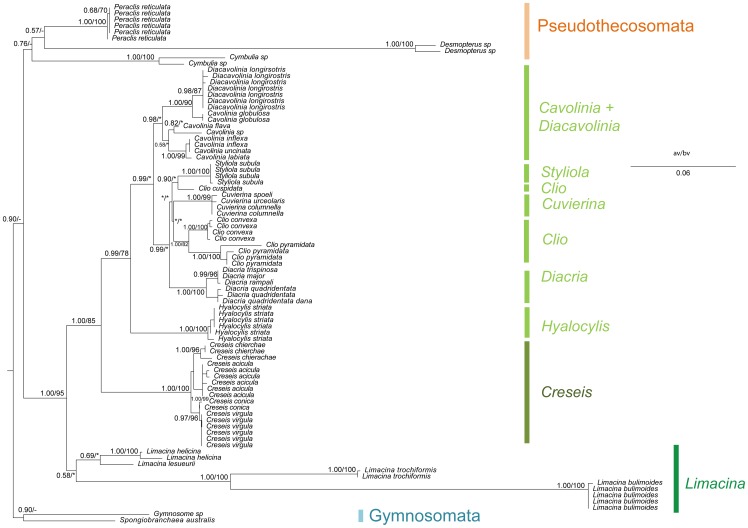
Phylogenetic analysis of Thecosomata based on partial 28S gene data set. We display the topology of the Bayesian tree issued from 28S gene data set without “noisy” site (888 base pair). For each clade, the maximum likelihood bootstrap values (bv) and the a-Bayes value (av) are indicated. Non supported group (av<0.5; bv<70%) are indicated by stars. Topological incongruences between a-Bayes tree and bootstrap tree are indicated by hyphens. Evolutionary rate is indicated by scale bar.

Considering the partial data set (without “noisy” sites, 888pb), we observed different phylogenetic relationships within Euthecosomata (Figure 6). The Creseidae were still paraphyletic with respect to *Hyalocylis* and *Styliola*. However, *Hyalocylis* was the sister group to the other genera (0.99/78) and not the sister group to *Cuvierina.* In addition *Styliola* was the sister group to *Cuvierina*, *Clio*, and *Diacria* genera (0.99/−). Interestingly, the phylogenetic position of *Hyalocylis* displayed “high” branch supports (bootstrap and aBayes) for this new topology while a polytomy was found for *Styliola*, *Cuvierina*, *Clio*, and *Diacria*. This lack of resolution is similar with the complete data set (with “noisy sites”) suggesting the limit of the 28S marker.

#### Divergence Time and Rates of Molecular Evolution

We estimated time divergence using the concatenated data set (i.e. 28S+COI based on 40 common sequences) with and without “noisy” sites (respectively 1670 and 1485 pb). Distances based method and Relaxed Bayesian method used the model that best fit the evolution of the whole data set, recognized as the selected COI model (i.e. for each codon position for the complete COI and one model for the COI without “noisy” sites) and the selected 28S model (one model for the complete 28S and one model for the 28S without “noisy” sites).

### 1: Method based on the distribution of pairwise genetic distance

*Limacina inflata* presented the most diverging substitution rate. The branch length test displayed four species with a substitution rate significantly different from the rest of the sequence data set (2 *Clio convexa*, 1 *Clio cuspidata*, 1 *Clio pyramidata* ). We conserved the concatenated data set without this four sequences and *Limacina inflata* for the rest of the pairwise genetic distance analysis. The smoothed frequency distributions of genetic distances were multimodal and were not significantly different from the null model (Birth and death process).

Four modes corresponding to high-density speciation events (Figure S1) were detected. The first one (Event 1: 59.1 Ma [46.9 , 114.2]) was consistent with the diversifying of the Gymnosomata, Pseudothecosomata and Euthecosomata, while the second (Event2: 37.7 Ma [23.8 , 46.9]) matched with the diversifying of Euthecosomata. Finally, the third event (Event 3: 19.7 Ma [8.1 , 23.8]) induced the diversifying of *Creseis*, *Diacria* and *Clio* species while the fourth event (Event 4: 2.4 Ma [0 , 8.1]) implied the diversifying of sister species in different genera or highly divergent lineage in a same species. Considering a molecular rate of evolution 4.6 10^−3^ subst/site three common nodes were found between the first speciation event (number of nodes  = 7) and the second (number of nodes  = 10), one between the second and the third (number of nodes  = 9), and none between the third and the fourth (number of node = 11) (correspondence between events and nodes are available in Figure S2).

Considering the complete data set (without “noisy” sites), three modes were detected (Event1: 46.8 Ma, Event 2: 28.8 Ma and Even 3: 2.2 Ma). The number of mode is different from result obtained with the complete data set (with “noisy” sites). This result is due to the fusion of two modes. Indeed, the mean (28.7 Ma) of Event 2 and Event 3 using the complete data set corresponded to the Event 2 of the partial data set (28.8 Ma). Removing “noisy” sites, that corresponded to gap alignment between divergent species belonging to two different Thecosomata families, induces a lost of phylogenetic information when considering two species belonging to the same genus. This bias impacts this methodology which is clearly sensitive to the lack of informative sites between closed species. For this reason, we will conserve the time divergence estimated using the complete data set (with “noisy” sites).

### 2: Relaxed Bayesian molecular clock

Considering the complete data set, all ESS values were above 150 and convergence on stationarity was quickly reached. The likelihood model of the two trees gives similar results. Indeed, the unconstraint tree displayed likelihood equal to −15117.01 ([−15140.69, −15095.20] 95% confidence interval [CI]) and the constraint tree displayed likelihood equal to −15117.09 ([−15138.76, −15096.13]). However the estimation of tree root was slightly different. The rate of substitution for the unconstraint tree was equal to 7.36 10^−3^ ([4.43 10^−3^–1.07 10^−2^] 95% confidence interval [CI]) for the 28S, 3.15 10^−2^ ([1.78 10^−2^–4.85 10^−2^]) for the first position of the COI gene, 7.13 10^−3^ ([2.66 10^−3^–1.33 10^−2^]) for the second position, and 1.13 10^−1^ ([6.74 10^−2^–1.70 10^−1^]) for the third position. The rate of substitution for the constraint tree was equal to 5.23 10^−3^ ([3.69 10^−3^–7.00 10^−3^] 95% confidence interval [CI]) for the 28S, 2.31 10^−2^ ([1.36 10^−2^–3.45 10^−2^]) for the first position of the COI gene, 5.12 10^−3^ ([2.04 10^−3^–9.91 10^−3^]) for the second position, and 7.85 10^−2^ ([4.89 10^−2^–1.10 10^−1^]) for the third position. Considering the partial data set (without “noisy” sites), the likelihood model displayed likelihood equal to −13025.44 ([−13047.16, −13001.59]. The rate of substitution was equal to 2.24 10^−3^ ([1.62 10^−3^–2.84 10^−3^] for the 28S, and 2.45 10^−2^ ([1.55 10^−2^–3.31 10^−2^] for COI.

The most important topological differences between unconstraint and constraint trees concerned the Euthecosomata monophyly both for analysis using the complete data set and the partial data set. Indeed, when Thecosomata monophyletic group was enforced, Euthecosomata were monophyletic while the Gymnosomata was the sister group to the Pseudothecosomata. Therefore, we favoured the result of time divergence analysis using constraint tree in this manuscript because the time calibration was based on the first split of Euthecosomata (56.5 Ma).

Divergence time for each node is presented in the constraint tree obtained with the complete data set (Figure S3) and partial data set (Figure S4). It is clear that Bayesian phylogenetic reconstruction is sensitive to “noisy” sites. Indeed, considering the analysis using the partial data set the phylogenetic position of the *Hyalocylis striata* species is in agreement with morphological tree in contrast of its position in the complete data set that is in accordance with the 28S gene tree. Furthermore, removing “noisy” sites (constituted by highly variable positions) had consequences on time divergence estimation and a tendency to increase them for the majority of nodes (e.g. divergence between *Creseis* and *Cuvierina* lineage was respectively 30.0 and 47.1 Ma).

## Discussion

### The monophyly of Euthecosomata species

A recent study based on COI gene tree [21] did not support the monophyly of the Euthecosomata although this clade was never questioned by previous studies since its establishment [12]. Our analysis also suggested the paraphyly of the Euthecosomata in COI phylogenetic tree. Indeed, two Euthecosomata taxa, *Hyalocylis striata* and *Limacina helicina*, constituted the sister group of Pseudothecosomata. However, this result conflicted with the 28S and the morphological trees, which strongly support the monophyly of Euthecosomata. Moreover such conflict in tree topologies was found for the *Cavolinia* genus for which the monophyly was not supported by COI trees unlike 28S and morphological trees.

According to paleontological knowledge, radiation of *Cavolinia* occurred in the early Miocene (e.g. *Gamopleura* [47], long after the first record from early Eocene of unwinding species such as *Camptoceros* (Wenz 1923) and the first straight shell species that was a *Creseis*-like fossil, and, or *Tibiella* (Meyer, 1884) and *Bucanoides* [44] two *Cuvierina*-like fossils from middle Ecocene [44]. This fossil record chronology conflicts with the COI tree in which *Cavolinia* branched before the divergence between *Creseis* and *Cuvierina* lineages. Moreover, according to the COI tree, the straight shell species could likely have appeared twice independently with a reversion in the lineage *Thilea helicoides*/*Limacina inflata* to explain the coiled shell observed in these species. However, the 28S/morphological trees did not support this scenario, which is the less parsimonious in terms of related morphological changes. Thus, it appeared that COI genes showed a lack of phylogenetic signal to infer Euthecosomata relationships. Similar conclusions have been drawn for others molluscs [48], [49], [50], [51] and more generally studies comparing the accuracy of mitochondrial and nuclear DNA sequences for phylogenetic analysis reporting that the latter is most informative for older divergence [52], [53], [54].

The hypotheses generally advanced for explaining incongruence between different gene trees are related to the intrinsic gene properties as well as their functions (protein-coding vs structural RNA) and the rate of evolution which is generally faster for the mitochondrial genes in many groups [55]. It is known that a high substitution rate saturates the phylogenetic signal by increasing homoplasy in mitochondrial genes such as COI. Such a saturation decreases the resolution of deep nodes of the phylogenetic tree [54]. The saturation process could be exacerbated by rate heterogeneity of substitution for COI gene within recognized mollusc classes [56], [57], [58]. This phenomenon produces a well-known artifact in phylogenetic reconstructions, the so-called “long-branch attraction artifact” [59]. However, if rate of substitution is heterogeneous among Euthecosomata, evolutionary models used for phylogenetic reconstructions normally tend to limit this effect [60], [37]. Thus, we assume that incongruence between gene trees for instance in *Hyalocylis* or *Limacina* does not result from a long-branch attraction artefact.

Incongruence between gene trees could also be the result of incomplete lineage sorting act [53], [61]. This makes such genes unsuitable for reconstructing phylogenies. However, this possibility seems unlikely for highly divergent taxa such as *Limacina* and *Hyalocylis* because numerous speciation events separate both lineages. Indeed, it is unlikely that two given lineages retain the same haplotype group after each speciation event. Such a case would imply a non-neutral evolution of the mitochondrial marker or an absence of lineage sorting. However, no sign of convergent evolution exists between mitochondrial DNA of *Hyalocilis* and *Limacina* that does not favour the lineage-sorting hypothesis.

Thus, heterogeneous rate of substitution and lineage-sorting effect do not alone explain the incongruence between the gene trees. Others hypothesis could be advanced such as hybridization phenomenon (e.g. [62], [63]), although so far inter-species hybridization has not been described in Thecosomata.

Furthermore, the monophyly of *Hyalocylis*+*Limacina* disappeared in the COI phylogenetic tree when partial data set was used, showing that the topology of COI tree could be influenced by “noisy” sites. Hence, the COI history could not reflect the « true tree » of Thecosomata, especially concerning deeper branching lineages. It seems more reasonable to favour the 28S tree that displayed high congruence with morphological and paleontological data for resolving the deep nodes. In the light of the reasons enumerated above, our analysis undoubtedly shows that Euthecosomata are monophyletic.

### Straight shell Euthecosomata: revival of Orthoconcha

Fol [18] previously proposed the term Orthoconcha to name all the straight shell species. Considering the first description of a straight shell specimen belonging to *Cavolinia* [64], this clade was named the Cavoloniidae Fisher, 1883. Although the taxonomic composition of Cavoliniidae underwent few changes, this nomenclature was followed by succeeding authors [9], [12], [65], [66], [13], [67]. However, some authors questioned the monophyly of straight shell species and suggested that the unwinding of the shell is a homoplasic state [17].

Based on 28S and morphological data, the straight shell species are monophyletic, a result which conflicts with the topology obtained with COI. However, the topology of the 28S and morphological tree is more reliable and congruent with the paleontological data (see above), our analysis provides more evidences for the existence of a clade consisting of all straight shell Euthecosomata, a clade also characterized by a second synapomorphy (#6 Helicoidal aragonitic microarchitecture of the shell). This hypothesis is the most parsimonious because it induces that the unwinding of the shell occurs only one time during Euthecosomata evolution, while according to the COI tree, the unwinding event is homoplasic and appears four times independently or two times considering one reversion step for *Limacina inflata* and *Thilea helicoides*. Thus, we propose to revive the term Orthoconcha firstly proposed by Fol [18] rather to re-establish the initial sense of Cavoliniidae from Pelseneer [9] for two reasons: 1) Orthoconcha refers as a synapomorphy of the group whereas Cavoliniidae refers to the *Cavolinia* genus, the species of which are characterized by one of the more derived shell state 2) the term Orthoconcha has never been modified and thus its definition could not lead to confusion conversely to Cavoliniidae, the definition of which is different according to the author considered.

### On the lack of consensus about traditional Euthecosomata families

Whatever the nomenclature used, none of the traditional families previously described has been confirmed except for Cavoliniidae, on the basis of morphological analysis (according to Rampal's nomenclature). The following discussion emphasizes the need to better define the boundaries between the traditional families of Euthecosomata.

#### Limacinidae

The monophyly of coiled shell Euthecosomata has rarely been questioned, from the initial grouping of these species into the *Limacina* genus by Gray [15]. The first change was proposed by Tesh [68] that replaced *Limacina helicoides* by *Thilea helicoides*. The second changes was proposed by Spoel [13] who determinated three sub-genus: *L.(Limacina) retroversa* and *helicina; L (Thilea) inflata, lesueurii* and *helicoides; L(Munthea) bulimoides* and *trochiformis.* Then, Rampal [14] proposed to split the Limacinidae into distinct genera: *Limacina* and *Thilea* excluded *Thilea helicoides* from Limacinidae. Wells [16] distinguished three main groups according to their reproductive mode. The first group defined by Wells is oviparous and consists of *L(Limacina) bulimoides*, *L. helicina, L. lesueurii, L. retroversa* and *L. trochiformis* while the second pseudo-viviparous and the third a placentary viviparous groups contains respectively *Limacina (Embolus) inflata (* =  *L. inflata*) and Limacina (*Thilea) helicoides*. Although deep branching in the COI tree cannot be resolved, the fact that *T. helicoides* and *Limacina inflata* are clearly isolated from the others *Limacina* favours the Wells hypotheses, in contrast to Van der Spoel view which is never supported by any gene or morphological tree.

In the present work, the monophyly of Limacinidae is supported neither by morphological nor by molecular data. Based on morphology Limacinidae are paraphyletic, a result which could be due either to a lack of adequate synapomorphy to define this clade (soft polytomy), or to the lack of a common ancestor (hard polytomy). Considering the molecular results, the COI tree is consistent with the analysis of Jennings et al. [21] who excluded *Thilea helicoides* from Limacinidae. The topology obtained unambiguously displayed two polyphyletic Limacinidae groups and the lack of common ancestor. Also the 28S analysis did not allow us to make a choice between a paraphyletic or polyphyletic assemblage of Limacinidae because of the lack of sequences for *T. helicoides* and *L. inflata.* As it is impossible to observe a synapomorphy for Limacinidae in the morphological tree when soft polytomy occurred (i.e. although they are monophyletic), none robust conclusion about the status of the Limacinidae can be drawn. According to Wells taxonomy, we propose *Embolus inflata* [16] instead of *Limacina inflata* to emphasize a putative soft polytomy, which occurs between *Limacina inflata* and others Limacinidae. Traditional Limacinidae are consequently split in three coiled shell genera *Limacina*, *Embolus* and *Thilea*, the phylogenetic relationships of which cannot be clearly determined.

#### Creseidae

This family was firstly proposed by Rampal [14] who clustered together three genera, *Creseis*, *Styliola*, and *Hyalocylis* which all exhibit a conical straight shell. However, the monophyly of Creseidae is not supported by the present work. The other taxonomic studies that grouped these three genera with *Clio* into the Clioinae [13], [67] are not supported either. Furthermore *Clio* never forms a monophyletic group with at least one of the three others genera considered. Moreover, there is a consensus from our analysis that places *Creseis* as the sister group to the all others straight shell species. Similarly, *Styliola* represents the sister group to Orthoconcha (at the exception of *Creseis*) based on morphological and 28S trees. Thus, none of the previous taxonomic hypothesis concerning *Creseis* and *Styliola* species is corroborated by our analysis and there is no argument for maintaining the Creseidae or Clioinae as valid clades. Owing to the fact that the most ancient straight shell fossils look like current *Creseis* shell (*Camptoceros*, [43] and *Creseis sp.* [44]), a conical straight shell is likely a plesiomorphic state in Orthoconcha. This hypothesis has been supported by different authors that considered *Creseis* as the less complex form among all the straight shell species [8]. Thus, Paleontological, morphological and molecular analysis leads us to suggest that Creseidae is not a natural taxon which once again illustrates the recurrent error of species grouping based on plesiomorphic states, as it is likely the case for the coiled shell Limacinidae.

#### Cavoliniidae

Cavoliniidae are recognized as the most ancient described family of straight shell species created after the first description of a specimen belonging to *Cavolinia* (see above). From its rise, this family was never contested but its taxonomic composition changed after the removal of several genera (*Clio* and the conical straight shell genera *Creseis*, *Hyalocylis* and *Styliola*) for creating new families (Creseidae, Clioidae or Cuvierinidae).

According to Rampal taxonomy, Cavoliniidae are represented by two subfamilies and four genera: the Cavoliniinae *Clio*, *Diacria* and *Cavolinia* and the Cuvierininae only represented by *Cuvierina.* First, based on COI tree, we observed some important topological differences due to the extreme divergence of *Hyalocylis* and *Cavolinia* sequences. Second, *Diacria*, *Clio* and *Cuvierina* genera were found monophyletic whatever the tree observed (COI, 28S and Morphology). Third, both morphological and 28S trees corroborate the monophyly of Cavoliniidae + *Hyalocylis*, and thus the belonging of *Clio* to Cavoliniidae. However, these trees are in conflict on two points i) Cavoliniinae (*Cavolinia*, *Clio*, *Diacria*) are monophyletic in morphological tree only and molecular tree implies that morphological innovation such as lateral ridges or lip aperture would be a convergence, that is unlikely ii) *Hyalocylis* is either the sister group to all others Cavoliniidae (morphological data) or the sister group to *Cuvierina* the 28S trees. According to the morphology, *Hyalocylis* diverges from Cavoliniidae before the first divergence inside the family, which separated *Clio* from *Cuvierina* 35.0 Ma. However, the 28S tree suggests a more recent divergence, dating after the divergence between *Diacria* and *Cuvierina* a result supported by paleontological data because the oldest *Diacria*-like fossil (*Diacrolinia*, Rang, 1827) record dates from early Miocene [47] whereas the first *Hyalocylis* record dates from late Miocene/early Pliocene [69].

It seems difficult to reassess rigorously the Cavoliniidae relationships because of the variable position of *Hyalocylis* and *Cavolinia* resulting of an absence of global congruence through the different molecular markers and the morphology. However, when “noisy” sites are removed, the positions of *Hyalocylis* are in congruence with the morphological tree favouring the hypothesis that *Hyalocylis* is the sister group of the Cavoliniidae. As a consensus, our results support the Cavoliniidae according to Rampal's definition [14] who claimed that *Clio* belongs to this clade but new molecular data will be required to confirm the position of *Hyalocylis* as the sister group of Cavoliniidae and the position of *Cavolinia* inside this family.

### Integrative approach of divergence time in Thecosomata lineage

Divergence time between Thecosomata lineages have been assessed by a molecular clock model based on pairwise genetic distance between sequences and a relaxed Bayesian molecular clock model. Those two models were performed respectively on the concatenated data set with “noisy” site and the concatenated data set without “noisy” site. The pairwise genetic distance based method was very sensitive to the quantity of site (best with “noisy” sites) attested by the loss of information related on diversifying event detected when data set without “noisy” sites is used. In contrast, relaxed Bayesian molecular clock model is sensitive to the quality of site (best without “noisy” sites) considering topological congruence with the morphological tree when partial sequences data set is used (i.e. *Hyalocylis* position as the sister group of Cavoliniidae). Moreover, divergence times were in general older and more congruent with paleontological data when “noisy” sites are removed (Table 3). In this way we considered this approach more robust for Bayesian model.

**Table 3:** Comparison of paleontological records, pairwise genetic distance based-method and relaxed molecular clock analysis (with/without “noisy” sites) for estimating time divergence.

Split Episode	Paleontology	Pairwise genetic distance based-method	Relaxed Bayesian Molecular Clock
1- Split between Euthecosomata and Pseudothecosomata	First Thecosomata: *Spirialis mercinensis* (Watelet & Lefèvre, 1885) 58 Ma ^a^ and First Pseudothecosomata fossil: *Altaspiratella* (Korobkov,1966) 56 Ma^b^	Event 1 (59.2 Ma)	58,6 Ma/57.3 Ma
2- Rising of the Orthoconcha	First *Creseis-*like fossil: *Camptoceros* (Wenz, 1923) 53 Ma^c^	Event 1 (59.2 Ma)	56.1 Ma/56.4 Ma
3- Rising of the Cavoliniidae	First *Cuvierina*-like fossil*: Bucanoides* (Hodgkinson, 1992) 50 Ma ^d^ *Tibiella* (Meyer, 1884) 50 Ma^d^	Event 1 (59.2 Ma)/ Event 2 (37.8 Ma)	30.0 Ma/47.1 Ma
4- Rising of the *Clio*	First *Clio* like-fossil:*Clio blinkae* (Janssen, 1989) 35 Ma^ e^	Event 2 (37.8 Ma)	22.6 Ma/29.7 Ma
5- Rising of the *Cavolinia*	First *Cavolinia-*like fossil: *Gamopleura* (Bellardi, 1873) 16 Ma^b^	Event 1 (59.2 Ma)/ Event 2 (37.8 Ma)	24.7 Ma/34.2 Ma
6- Rising of thre *Diacria*	First *Diacria*-like fossil: *Diacrolinia* (Rang, 1827) 21 Ma^b^	Event 2 (37.8 Ma)/ Event 3 (19.8 Ma)	18.2 Ma/26.5 Ma
7- Rising of the *Hyalocylis*	First *Hyalocylis* like-fossil: *Hyalocylis* *haitensis* (Collins, 1934) 6 Ma^f^	Event 1 (59.2 Ma)/ Event 2 (37.8 Ma)	16.1 Ma/38.5 Ma

The table showed the time divergence estimation of 7 putative split episodes that occurred during Thecosomata evolution. Paleontological estimates correspond to the oldest fossils record found by different authors: a = [104], b =  [47], c =  [43], d =  [44], e = [79], f = [69]. The time divergence of Event 1 is estimated at 59.1 [46.9, 114.2] Ma, the event 2 at 37.7 [23.8, 46.9] Ma, the event 3 at 19.74 [8.1, 23.8] Ma and the event 4 at 2.4 [0,8. 1] Ma. The two values presented for the relaxed clock analysis correspond respectively to the values obtained with complete data set (with “noisy” sites) and partial data set (without “noisy” sites).

The divergence time of 7 major putative events among the current Thecosomata lineages presented in Table 3 were congruent between the two models and were corroborated by paleontological data excepting for three Split Episodes.

Split Episode 4 corresponds to the rising of the *Clio* lineage. In other words, when did the first fossil with lateral ridges appeared? This period corresponds to the second diversifying event (37.8 Ma) of the pairwise distance based method, which contains the divergence between all the Cavoliniidae lineages that are *Cuvierina*, *Clio*, *Diacria* and *Cavolinia*. This is supported by paleontological data indicating that the first fossil with lateral ridges on the shell dates from the Rupelian (33.0 Ma) and looked like *Clio*. The divergence time assessed by the Bayesian approach under-estimated slightly the divergence time of the first split of Cavoliniidae (29.7 Ma).

Split Episode 5 corresponds to the rising of *Cavolinia*. The two molecular estimations are congruent. Indeed, the pairwise distance based method indicated an emergence around 37.8 Ma and for the Bayesian method 34.2Ma. This result conflicts with the paleontological data because the first *Cavolinia*-like fossil (*Gamopleura*) was recorded from the early Miocene (nearly 16.0 Ma). The assumption that the first *Cavolinia*-like fossil have emerged around 20 Ma after the rising of *Cavolinia* lineage could explains this incongruence. However, as we discuss above, the resolution of the two genes did not allow to decipher about the phylogenetic relationships between Cavoliniidae. Therefore, the position of *Cavolinia* using the concatenated data set (that is also incongruent with morphological tree) is questionable and it is likely that the molecular time divergence estimations of *Cavolinia* lineage were over-estimated due to a “wrong” position.

Split Episode 7 corresponding to the rising of *Hyalocylis*. Similarly of the Split Episode 5, the two molecular estimations conflicts with the paleontological data displaying that the first *Hyalocylis*-like fossil recorded from the late Miocene (nearly 6.0 Ma). However, in this last case, we can hypothesize that *Hyalocylis*-like morphology emerged long time after the rising of *Hyalocylis* lineage because its topological position in the relaxed Bayesian molecular clock model is in agreement with the morphological tree.

### Evolutionary scenario based on morphology, molecules and paleontological data and implication for body plan novelties

#### First diversifying event

**Figure 7:**
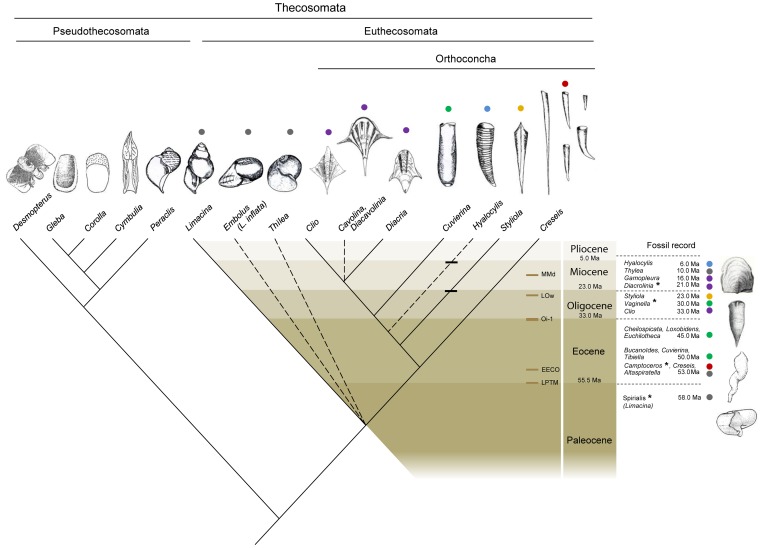
Evolutionary scenario of Thecosomata. On the right is denoted a series of fossil records. The names of the four drawn fossils are indicated by asterisks. Colors grouped fossils and living species together according to their closed morphology. The dotted lines characterize the unresolved branching:. Five paleoclimatic events mentioned in the text are also indicated and correspond to Late Paleocene Thermal Maximum (LPTM), Early Eocene Climatic Optimum (EECO), Oi-1 Glaciation (Oi-1), Late Oligocene warming (LOw) and Middle Miocene disruption (MMd) that corresponded to the Langhian/Serravalian boundary. Paleontological estimates correspond to the oldest fossils record from different studies: [103] for *Thilea*; and [44] for *Vaginella* Daudin, 1800 *Cheilocuspidata* Hodgkinson, 1992, *Loxobidens* Hodgkinson, 1992 and for *Euchilotheca* Fisher, 1882. See Table 3 for the others fossil references. The older *Styliola* and *Hyalocylis*-like fossil was represented by horizontal line on the branch.

The abundance of *Limacina*-like fossils recorded just after the Cretaceous/Tertiary mass extinction suggests that a radiation occurred from a benthic mollusc ancestor to a morphology more adapted to planktonic lifestyle [44]. This main radiation rise to the Euthecosomata and Pseudothecosomata lineages (Figure 7), both characterized by a coiled shell ancestor, a shell morphology observed in many benthic gastropods. Concerning the Euthecosomata lineage, a hypothetical *Limacina* ancestor likely split in several lineages, giving rise to the current Limacinidae recognized as *Limacina sensu stricto*, *Embolus, Thilea* and the Orthoconcha ancestor. Considering the fact that a partially unwinding fossil belonging to *Camptoceros* and *Creseis sp* dated from nearly 53.0 Ma (Early Eocene), the unwinding of the shell leading to Orthoconcha likely occurred quickly in a range of 3–4.0 Ma. The emergence of the Orthoconcha occurred in the context of important turn-over in marine planktonic community due to severe environmental changes as global warming, marine oligotrophication and ocean acidification [70], [71], [72], [73], [74], that started from the Late Paleocene Thermal Maximum event currently dated at ∼55.5 Ma [75].

#### Second diversifying event

Morphological and molecular data showed that *Creseis* is the sister group to all others Orthoconcha. In addition, *Creseis*-like is the first Orthoconcha in fossil records. Hence, there is a bundle of evidence that the primitive *Creseis* morph (i.e.: conical shell) corresponds to the last common ancestor of Orthoconcha. According to this scenario, the conical straight shell observed in *Creseis* underwent a developmental repatterning resulting in a partially dorso-ventrally depressed shell, a morphology observed in adults of *Cuvierina*. This evolutionary sequence is corroborated by the fact that *Cuvierina* juveniles exhibit a typical conical shell, a state which corresponds to the plesiomorphic shape of their *Creseis*-like ancestor. This lineage have diverged from the *Creseis* lineage following a second hyperthermal events (The Early Eocene Climatic Optimum) that occurred between 53.0 and 50.0 Ma depending of authors [76], [77] and [78]. Then, these two lineages flourished from this time with a peak of diversity (e.g. *Creseis*, *Bucanoides, Tibiella, Cuvierina, Euchilotheca*, *Loxobidens*) during the Bartonien (Middle Eocene) that preceded a diversity collapse during the late Eocene [44] and [47]. From this time, three current lineages diverged from a *Cuvierina*-like ancestor. The *Styliola* genus could be the first rising lineage from a *Cuvierina*-like ancestor and displayed its actual morphology about 24.0 Ma ago [47] as a lower bound. Because of the conical shell of *Styliola,* which have similar juvenile shell shape and protochonch of *Cuvierina* morph, we suspected that *Styliola* resulted of a neothenic process. In a similar way, *Hyalocylis* lineage diverged from *Cuvierina* lineage during the Eocene and displayed its actual morphology about 6.0 Ma ago. The third diversifying event from the *Cuvierina* lineage rise to the lineage characterized by the lateral ridges (#10) resulting of the dorso-ventraly depression of the whole teloconch (which is restricted to the anterior part in *Cuvierina)*. This event is illustrated by the oldest fossil that exhibited partial lateral ridges such as *Vaginella* and the first *Clio*-like fossil found during the Rupelian (Early Oligocene), that followed an important turn-over of Thecosomata species [79] correlated with the Oi-1Glaciation event (∼33.0 Ma, [76]), that marked the Eocene/Oligocene boundary.

#### Third diversifying event

Late Oligocene is marked by the diversification of the species with complete dorso-ventrally depressed shell such as *Clio* and *Vaginella* fossils (e.g. *Clio vasconiensis,* [47]). These diversifying events occurred in the context of the Late Oligocene Warming (26–24.0 Ma), a short but intense warming and acidification of the ocean [76], [80] correlated with planktonic community changes (e.g. [81]) and rich assemblages deposition of calcareous organism including Thecosomata (see references *in* [82], [83], [84], [85], [47]). This diversifying event within *Clio* lineage has led to the *Cavolinia* and *Diacria* lineages during the early Miocene, both characterized by the presence of lips around their shell aperture. Fossil-species such as *Diacrolinia* illustrated the rising of these new lineages that are described as the intermediary form between *Diacria* and *Cavolinia* as between *Clio* and *Diacria* because of the thickened aperture of the lips [86]. The diversity of complete dorso-ventrally depressed shell fossil (i.e. *Vaginella, Clio*, *Diacrolinia*, *Diacria*, *Gamopleura*, *Cavolinia*) and the molecular divergence time leading to the current intra-genus diversity of *Clio*, *Cavolinia*, and *Diacria* (i.e. Diversifying event 3: 19.8 Ma) shows that these lineages diversified from early Miocene until a species-diversity peak that was recorded during the Langhian (Middle Miocene). This time was characterized by a warming period leading to profound trophic re-organization in ocean attested by the decline of carbonate-producers phototrophs [87]. This diversity collapsed during the Middle Miocene (i.e. from the Langhian/Serravalian boundary) [47] that marked the start of a long term cooling period and important sea-levels variations [88], [76].

#### Fourth diversifying event

From the Middle Miocene the marine temperature declines [89], [76] and molecular clock showed that for certain lineages intra-species polymorphism originated from a fourth event, as it is the case for *Creseis virgula*, *Clio pyramidata* and *Peraclis reticulata* lineages.

#### Shell evolution and the role of predation pressure and buoyancy

Abundant bibliography illustrates the strong influence of predation on shell morphology in Gastropoda (e.g.[90], [91]). Several studies have shown that Thecosomata are subject to important predation by other planktonic organism such as Gymnosomata [92], [93], [94] or by Thecosomata themselves that can lead to important events of cannibalism [92]. [94] argued that Gymnosome and Thecosomata co-evolved in a prey-predator system which induced some morphological evolutions which can be discussed in this sense. However, the paradox of the first morphological evolution is that the unwinding of the shell is correlated with the loss of the opercula in the Orthoconcha, a clear defensive feature that can act as a barrier to digestion in mollusc [88], [91]. This loss was compensated by the conical shape in *Creseis*-like ancestor that improves their escape capabilities during predation events by optimizing the rate of descent through the water column [95]. In the *Cuvierina*-like species, it is compensated by a dorso-ventrally depression in the anterior part of the telochonch, narrowing the aperture that is considered as a typical anti-predatory adaptation in mollusc [91]. This tendency was enhancing in *Cavolinia* and *Diacria* lineage with the innovation of lip, that is considered as a shield-like protection of the peristoma [96].

Although *Creseis* exhibited a spectacular escape strategy with their conical straight shell, predation pressure appears to be insufficient to explain the unwiding of the shell (implying the loss of opercula) and the complete dorso-ventrally depression which first appeared on *Clio*-like organisms. As it was hypothesized for the ammonite [97], [98], [99], we can argue that the unwinding of the shell should optimize the energy dispenses for locomotion by the transition from an helical swim, observed in *Limacina* species [100], to a more rectilinear swim as seen for *Creseis* [95]. Later, the locomotion performance was improved with the innovation of lateral ridges and their extension (i.e. lateral spines) that increased the surface/volume balance, and thus the buoyancy of the shell. Attested by the diversity of complete dorso-ventrally depressed fossil, this evolutionary tendency was increased during warm periods that spread during the late Oligocene to mid-Miocene. Therefore, this correlation jeopardized the role of ocean temperature and water mass density changes that might favor more buoyant shell during warmer periods.

Hence, the radiation of Thecosomata emerged in the context of the “planktonic ecospace” release after the Cretaceous-Tertiary. From this switching of benthic life-style to the planktonic life-style, predation and shell buoyancy seems to have played a major role in the diversifying of Thecosomata, that were rhythmed by climatic changes and species turn-over that spread from the Eocene to Miocene.

## Conclusion

Our results corroborated the consensus from previous taxonomical studies concerning the monophyly of Euthecosomata and Pseudothecosomata. However, the present study implies changes of the Euthecosomata classification, which could be considered as a mix between the previous one. We showed that the main changes concerned the taxa that are based on a plesiomorphic character, such as Limacinidae and Creseidae. In order to complete the taxonomy of Thecosomata, future works must be conducted to establish 1) the phylogenetic position of the three genus that constituted the Limacinidae 2) to define the relationships between Cavoliniidae species 3) a new taxonomic nomenclature that consider the taxonomic group represented by only one genus that are *Creseis* and *Styliola* lineages. In lower taxonomical scale, we encourage studying species-relationship because it is expected that phenotypic plasticity [101] and cryptic species [102] could have biased the rank assignations of the taxonomical entities.

The present study brings also new insight on the morphological evolution in Thecosomata. For the first time, we showed the monophyly of the Orthoconcha, suggesting that the unwinding of the shell appeared once in the lineage of living straight shell species. Moreover, the monophyly of Cavoliniidae led us to conclude that a straight shell lineage derived from a *Cuvierina*-like ancestor, even for *Styliola*, which is characterized by plesiomorphic shell state (conical shape). Therefore, we conclude that Euthecosomata evolution is driven by a combination of evolutionary novelties (e.g. unwinding of shell, teloconch differentiation, lateral ridges), and morphological reversion for instance in *Styliola*.

## Acknowledgments

We would like to acknowledge people involved during sampling of specimens of the different expeditions. We particularly thank Gabriel Gorsky and all the consortium members who sampled during three years of TARA Oceans expedition and l′Observatoire Océanologique de Villefranche sur Mer (UPMC Sorbonne Universités/CNRS, France) in this study. The author deeply appreciate the following institution and their ship time support for the sampling dedicated to molecular analysis: l′Observatoire Océanologique de Villefranche sur Mer (Université Paris VI, France), El Colegio de la Frontera Sur (ECOSUR) Centre d'Océanologie de Marseille, Institut de Recherche pour le Development de Polynésie française (IRD Tahiti). The samples used during the morphological analysis originated from expedition of the following institution: Institut Scientifique et Technique des Pêches maritimes (Président-Théodore-Tissier and Thalassa ships), Marinbiologisk Laboratorium of Charlottenlund Slot, Danemark (Thor and Dana ships), Office de la recherche Scientifique et Technique d'Outre-mer, Centre de Nouméa (Coriolis ship), Instituto de Investigaciones Pesqueras, Barcelona (Magga-Dan ship), Alexandria University (Shoyo-Maru ship), Centre National pour l′Exploitation des Océans (Jean-Charcot ship). Thanks also Marc Pagano for its precious samples from French Polynesia, Gabriel Nève for his logistical help during specimen diagnosis, Jonathan Bonet for his useful comments and Céline Jouenne for proofreading the article. Finally, thank to Dr. Dirk Steink and two anonymous referees for their valuable comments and their constructive suggestions that improve considerably the manuscript. All authors read and approved the final manuscript. This article is the contribution no. 02 of the Tara Oceans Expedition 2009/2012.

## Supporting Information

Figure S1 Pairwise genetic distance densities and time divergence estimated. The smoothed distributions of corrected pairwise distances between sequences from the concatenated set, the 28S gene and the COI gene are indicated in red. Distributions of pairwise distances obtained from 1000 simulated H_0_ distributions (Birth-death model) are in thin gray, and their mean distribution in thin black. *p*-value of the corresponding test is also indicated for each data set. X-axis corresponds to time divergence estimation corresponded of pairwise genetic distances using the estimated molecular substitution rate (4.6 10^−2^subst/site); y-axis corresponds to their densities. Four modes indicated by red arrows are observed in the concatenate data set corresponding of four diversifying events.
(TIF)

Figure S2 Time divergence estimated by the pairwise genetic distance based method. The neighbourg-joining trees are based on the concatenated (COI and 28S) data set and illustrates by red circle the nodes concerned by one of the four diversifying events and the concerned lineage by red lines. The x-absiss corresponds to the genetic distance from the hypothetical common ancestor (dist = 0).
(TIF)

Figure S3 Estimates of time divergence by the Relaxed Bayesian molecular clock based on the concatenate complete data set (657 bp for COI and 1013 bp for 28 S). Divergence time in Ma estimates are indicated under branches, and 95% credibility intervals are represented as gray bars centered on the nodes. The thicknesses of branches are proportionated to the evolutionary rate estimated. Time divergence was indicated by a scale bar in Ma. Noted that it is the constraint tree for which the monophyly of Euthecosomata was forced.
(TIF)

Figure S4 Estimates of time divergence by the Relaxed Bayesian molecular clock based on the concatenate partial data set (607 bp for COI and 888 bp for 28 S). Divergence time in Ma estimates are indicated under branches, and 95% credibility intervals are represented as gray bars centered on the nodes. The thicknesses of branches are proportionated to the evolutionary rate estimated. Time divergence was indicated by a scale bar in Ma.Noted that it is the constraint tree for which the monophyly of Euthecosomata and Orthoconcha was forced.
(TIF)

Table S1 List of morphological character and coding information.
(DOCX)

Table S2 Morphological data matrix Character list and character state code is available on Table S1. Unknown character states are indicated by a question mark and non-homologous characters are indicated by an asterisk.
(DOCX)

Table S3 Models of mtDNA (Co1) and Nuclear (28S) sequence evolution. The best models were estimated using the Bayesian Information Criterion (BIC).
(XLSX)
