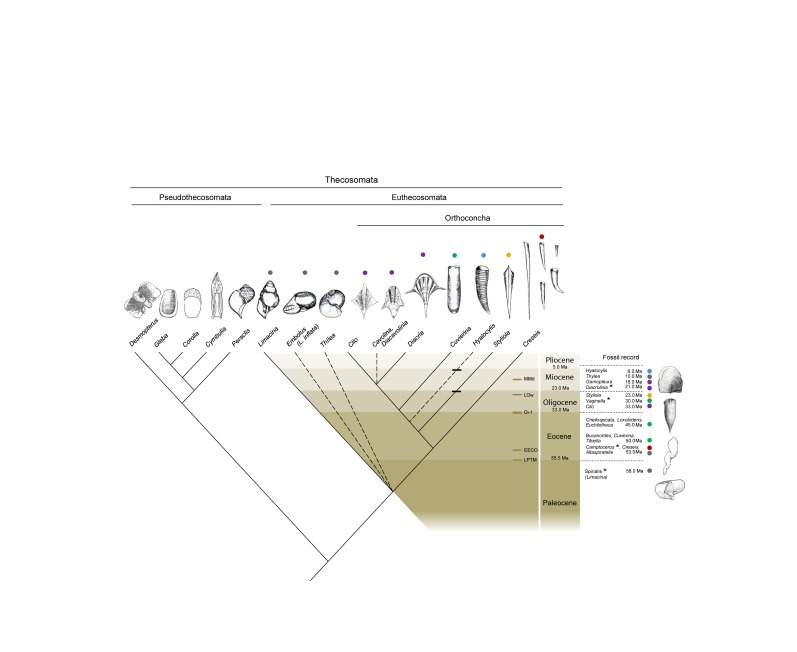# Correction: Phylogenetic Analysis of Thecosomata Blainville, 1824 (Holoplanktonic Opisthobranchia) Using Morphological and Molecular Data

**DOI:** 10.1371/annotation/6f40430d-0e3e-48ba-8a31-49cb4e52dd0d

**Published:** 2013-11-15

**Authors:** Emmanuel Corse, Jeannine Rampal, Corinne Cuoc, Nicolas Pech, Yvan Perez, André Gilles

In Figure 7, there is an error related to the schematized shell morphology and genus correspondence. The shell scheme corresponding to Cavolonia and Clio genus was inverted. Please see the corrected Figure 7 here: 

**Figure:**