# Supplementary figures and images for: Phylogenetic Analysis of Thecosomata Blainville, 1824 (Holoplanktonic Opisthobranchia) Using Morphological and Molecular Data
Source: PLoS One. 2013 Apr 12;8(4):e59439. doi: 10.1371/journal.pone.0059439 (PMC3625178; doi:10.1371/journal.pone.0059439)

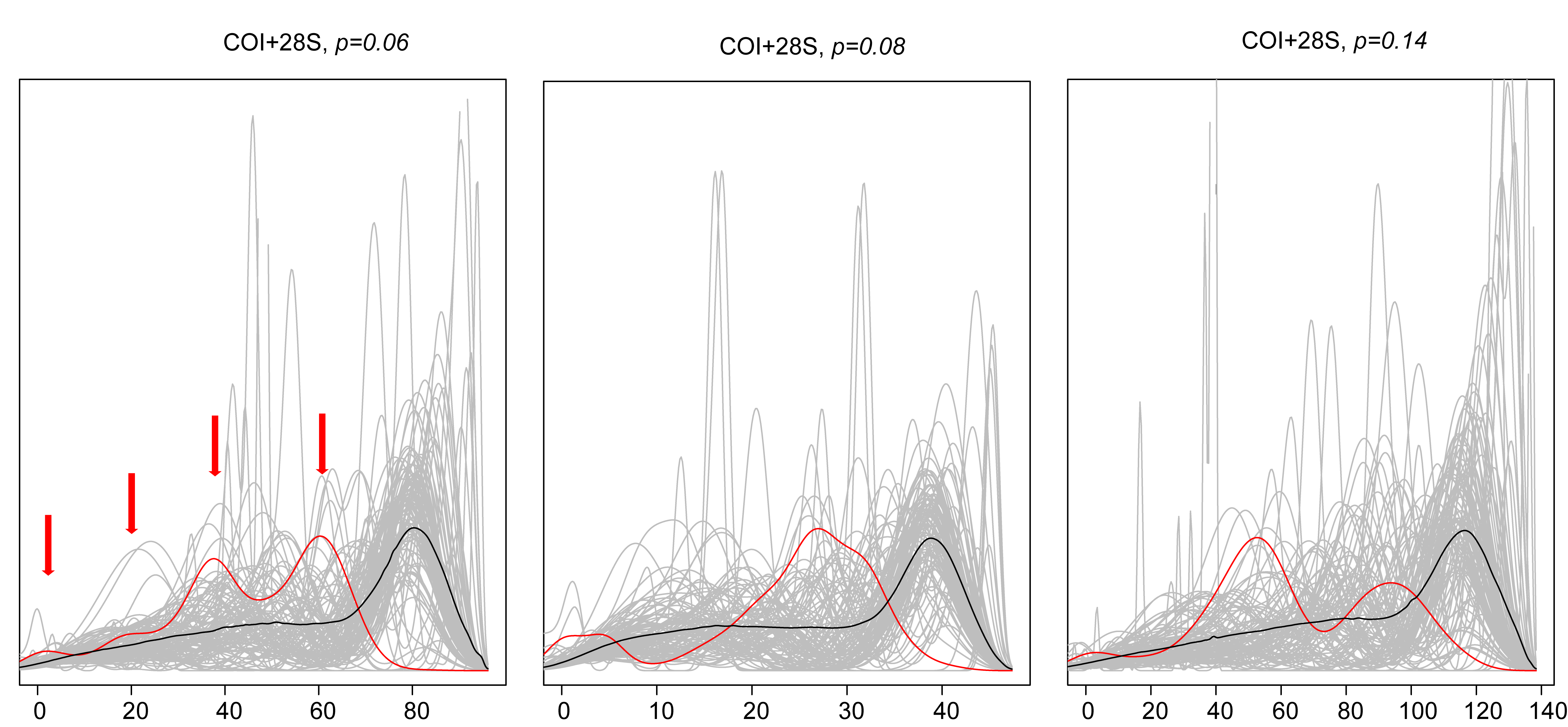

Supplement: Figure S1 — Pairwise genetic distance densities and time divergence estimated. The smoothed distributions of corrected pairwise distances between sequences from the concatenated set, the 28S gene and the COI gene are indicated in red. Distributions of pairwise distances obtained from 1000 simulated H0 distributions (Birth-death model) are in thin gray, and their mean distribution in thin black. p-value of the corresponding test is also indicated for each data set. X-axis corresponds to time divergence estimation corresponded of pairwise genetic distances using the estimated molecular substitution rate (4.6 10−2subst/site); y-axis corresponds to their densities. Four modes indicated by red arrows are observed in the concatenate data set corresponding of four diversifying events. (TIF) [file pone.0059439.s001.tif]

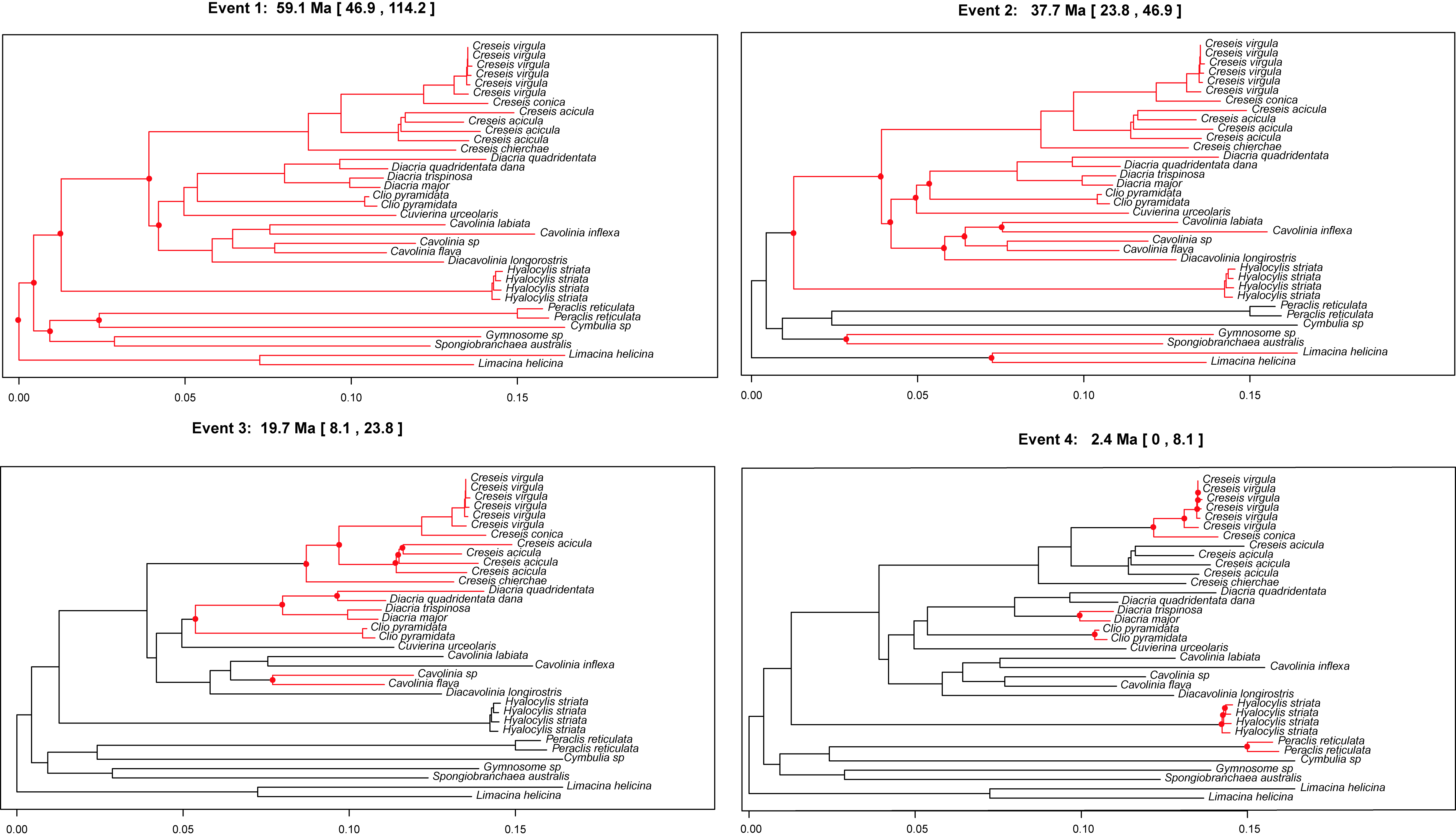

Supplement: Figure S2 — Time divergence estimated by the pairwise genetic distance based method. The neighbourg-joining trees are based on the concatenated (COI and 28S) data set and illustrates by red circle the nodes concerned by one of the four diversifying events and the concerned lineage by red lines. The x-absiss corresponds to the genetic distance from the hypothetical common ancestor (dist = 0). (TIF) [file pone.0059439.s002.tif]

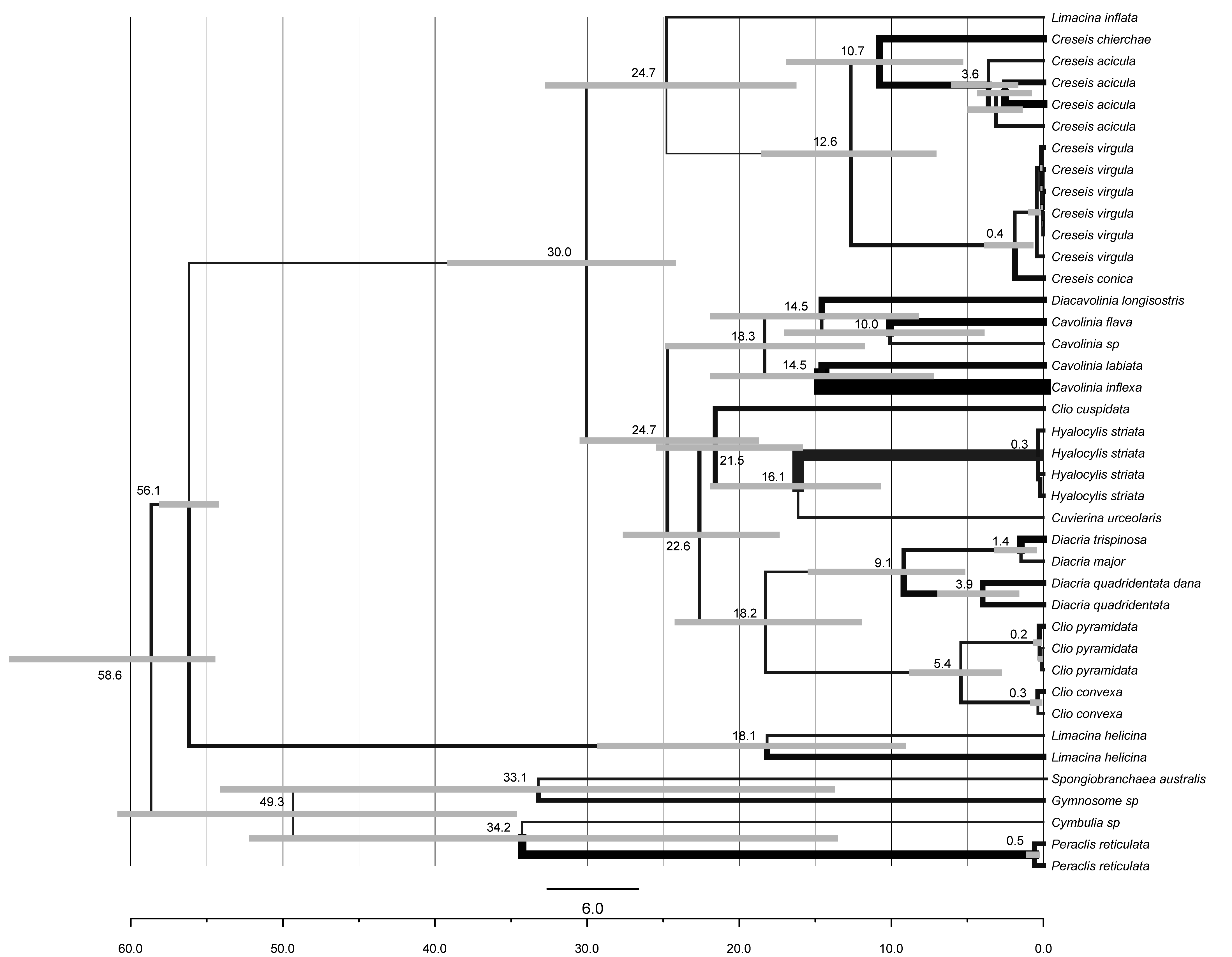

Supplement: Figure S3 — Estimates of time divergence by the Relaxed Bayesian molecular clock based on the concatenate complete data set (657 bp for COI and 1013 bp for 28 S). Divergence time in Ma estimates are indicated under branches, and 95% credibility intervals are represented as gray bars centered on the nodes. The thicknesses of branches are proportionated to the evolutionary rate estimated. Time divergence was indicated by a scale bar in Ma. Noted that it is the constraint tree for which the monophyly of Euthecosomata was forced. (TIF) [file pone.0059439.s003.tif]

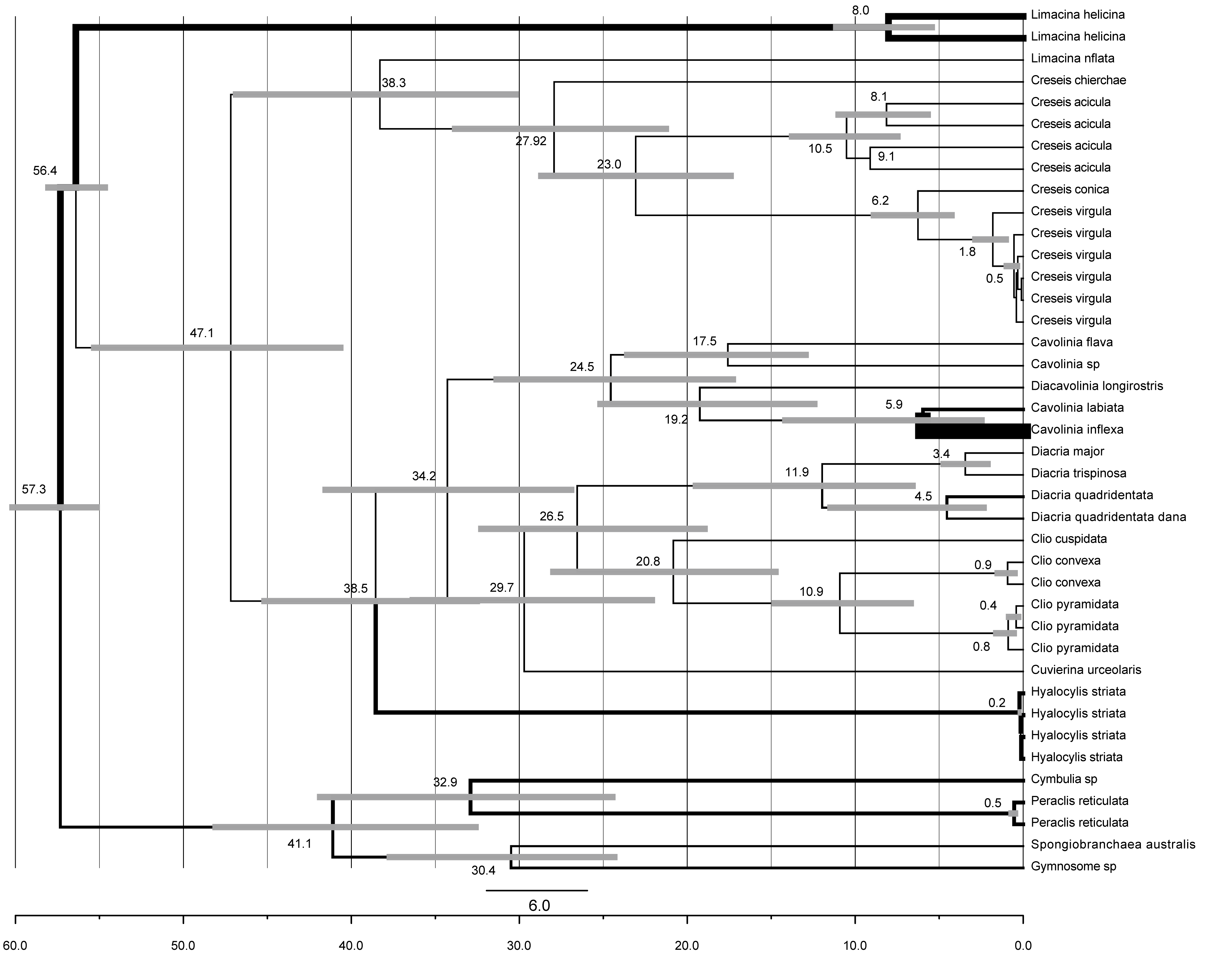

Supplement: Figure S4 — Estimates of time divergence by the Relaxed Bayesian molecular clock based on the concatenate partial data set (607 bp for COI and 888 bp for 28 S). Divergence time in Ma estimates are indicated under branches, and 95% credibility intervals are represented as gray bars centered on the nodes. The thicknesses of branches are proportionated to the evolutionary rate estimated. Time divergence was indicated by a scale bar in Ma.Noted that it is the constraint tree for which the monophyly of Euthecosomata and Orthoconcha was forced. (TIF) [file pone.0059439.s004.tif]
