# Supplementary material for: Phylogenetic Analysis of Thecosomata Blainville, 1824 (Holoplanktonic Opisthobranchia) Using Morphological and Molecular Data
Source: PLoS One. 2013 Apr 12;8(4):e59439. doi: 10.1371/journal.pone.0059439 (PMC3625178; doi:10.1371/journal.pone.0059439)
Supplement: Table S1 — List of morphological character and coding information. (DOCX) [file pone.0059439.s005.docx]

**SHELL AND PSEUDOCHONCA**

C1- Shell or pseudochoncha : Absent=0 ; Present=1.

C2- Calcareous shell (telonchonca, juvenile shell, protochonca)=0; aminated pseudochoncha=1.

C3- External shell=0; inner pseudochoncha=1.

C4- Pseudochoncha: ovoid=0; long=1.

C5- Left spiraled shell=0; symmetrical straight shell=1.

C6- Straight telochoncha: aragonitic microarchitectural structure: prismatic or crossed-lamellar=0; helicoidal=1.

C7- Spiralled shell. Columnella and clear microsculpture: absent=0; present=1; pseudocolumnella and microsculpture absent=2.

C8- Straight teloconcha: transversal cross-section nearly circular=0; triangular=1; lenticular=2; oval=3.

C9- Straight telochoncha without lateral ridges: morphology. Conical with longitudinal oblique groove=0; conical without oblique groove=1; conical dorso-ventraly depressed with transversaly striations=2; bottle shaped with kidney-shape aperture=3.

C10- Straight and dorso-ventraly depressed telochoncha with lateral ridge morphology: pyramidal telochoncha with lateral ridge, wide aperture without differenciated lip=0; more or less globular telochoncha with lateral ridge, slit and confined aperture with differenciated lips=1.

C11- Straight telochoncha: lateral ridges. Shaped =0; flattened=1.

C12- Straight telochoncha: lateral ridges. Short=0; long spines=1.

C13- Straight telochoncha: extremity of lateral slits. Sharp=0;wide=1.

C14- Straight telochoncha: longitudinal dorsal ribs: number.

C15- Straight telochoncha: longitudinal groove on the dorso-lateral ribs. Absent=0; present=1.

C16- Straight telochoncha:medio-dorsal rib. Short and smooth=0; long and acute=1.

C17- Straight telochoncha: Catch system of dorsal and ventral side. Absent=0; present and simple=1; present and double=2.

C18- Straight telochoncha: dorsal lips. Thin=0; Thickined=1.

C19- Protochoncha. Little apparent =0; truncated=1; differentiated=2.

C20- Straight telochoncha: juvenile shell. Absent=0; present and persistent=1; present and discarded=2.

**WING-SHAPED FOOT SYSTEM**

C21- Swimming disc. With indent =0; joint=1.

C22- Swimming disc: Ventral lobe. Absent=0; present=1.

C23- Swimming disc: mucus gland. Absent=0; present=1.

C24- Two parapodia. Bilobed=0; one-lobed=1; one-lobed swimming plate shaped=2.

C25- Parapodia: tentacular lobe on the dorsal edge. Absent=0; present=1.

C26- Parapodia: ciliated zone near the basis. Absent=0; present=1.

C27- Posterior foot lobe: form. Varied=0; half-circular=1.

**CEPHALIC ZONE**

C28- One compact cephalic lobe=0; Two little proeminence=1.

C29- Proboscis. Absent=0; present and joint to the parapodial disc=1; present and half free=2; present and free=3; pseudoproboscis=4.

C30- Rhinophores. Asymmetrical=0; symmetrical=1.

**PALLIAL SYSTEM**

C31- Pallial cavity present: dorsal=0; ventral=1; dorso-ventral=2; right-lateral=3. Pallial cavity absent=4.

C32- Pallial gland very expanded. Zones of differents cells: number. One zone=0; two zones=1; three zones=2; five zones=3. Pallial gland reduced=4.

C33- Pallial gland: parallalelepipedic cells: number of zones. Zero zone=0; one zone=1; two zones=2 ; three zones=3.

C34- Pallial gland: parallalelepipedic cells: type. Simple=0; simple and crateriform=1.

C35- Pallial gland: majority cells. Prismatic=0, parallalelepipedic=1; prismatic and parallalelepipedic=2.

C36- Pallial gland: dum-bells shaped prismatic cells zone. Absent=0; present=1.

C37- Pallial cavity: anterior free side. Symetric double fringe=0; asymmetric double fringe=1; symmetric unifringe=2.

C38- Pallial cavity, anterior free side: deciduous leaf shaped appendix. Absent=0; present=1.

C39- Dorsal edge: medio-dorsal appendix. Absent=0; present=1.

C40- Lateral edge. Two fringes=0; Three fringes=1.

**RESPIRATORY SYSTEM**

C41- Conjonctive membrane=0; rudimentary feather-shaped gill=1; ctenidie=2; pectiniform gill=3.

**ALIMENTARY SYSTEM**

C42- Intestine. One buckle=0;other modality=1.

C43- Jaws. Absent=0; present=1.

C44- Radula. Absent=0; present=1.

C45- Radula. Serrated edge with big tooth=0; serrated edge with little tooth=1.

C46- Radula: Additional lateral tooth. Absent=0; present=1.

C47- Radula: lateral tooth. One adge serrated=0; two edge serrated=1.

C48- Radula: lateral and median teeth. Equal=0; different=1.

**GENITAL SYSTEM**

C49- Embryonic development. Oviparity=0; aplacentary vivparity=1; pseudoviviparity=2.

C50- Genital orifices. One orifice=0; two orifices.

C51- Spermatophores. Absent=0; present=1.

C52- Seminal groove. Present=0; absent=1.

C53- Albumine and mucus gland, penis. Absent=0; present=1.

C54- Deciduous cervical organ. Absent=0; present=1.

**NERVOUS SYSTEM**

C55- Visceral ganglia. Three ganglia (two symmetric lateral ganglia)=0; two asymmetric ganglia=1.
