# Supplementary material for: Phylogenetic Analysis of Thecosomata Blainville, 1824 (Holoplanktonic Opisthobranchia) Using Morphological and Molecular Data
Source: PLoS One. 2013 Apr 12;8(4):e59439. doi: 10.1371/journal.pone.0059439 (PMC3625178; doi:10.1371/journal.pone.0059439)
Supplement: Table S2 — Morphological data matrix Character list and character state code is available on Table S1. Unknown character states are indicated by a question mark and non-homologous characters are indicated by an asterisk. (DOCX) [file pone.0059439.s006.docx]

***Limacina bulimoides*** 1 0 0 * 0 0 0 * * * * * * * * * * * 0 * * * * 1 0 0 0 0 0 0 0 2 2 0 0 0 2 0 0 * ? 0 1 1 0 0 0 1 0 0 0 0 1 0 1***Limacina helicina*** 1 0 0 * 0 0 0 * * * * * * * * * * * 0 * * * * 1 1 0 0 0 0 0 0 2 2 0 0 0 2 0 0 * ? 0 1 1 0 0 0 1 0 0 ? 0 1 0 1***Limacina inflata*** 1 0 0 * 0 0 0 * * * * * * * * * * * 0 * * * * 1 0 0 0 0 0 0 0 2 2 0 0 0 2 0 0 * ? 0 1 1 0 1 0 1 2 0 1 0 0 0 1***Limacina lesueuri*** 1 0 0 * 0 0 0 * * * * * * * * * * * 0 * * * * 1 0 0 0 0 0 0 0 2 2 0 0 0 2 0 0 * ? 0 1 1 0 0 0 1 0 0 0 0 1 0 1***Limacina retroversa*** 1 0 0 * 0 0 0 * * * * * * * * * * * 0 * * * * 1 1 0 0 0 0 0 0 2 2 0 0 0 2 0 0 * ? 0 1 1 0 0 0 1 0 0 0 0 1 0 1***Limacina trochoformis*** 1 0 0 * 0 0 0 * * * * * * * * * * * 0 * * * * 1 0 0 0 0 0 0 0 2 2 0 0 0 2 0 0 * ? 0 1 1 0 0 0 1 0 0 0 0 1 0 1***Thilea helicoides*** 1 0 0 * 0 0 2 * * * * * * * * * * * 0 * * * * 1 0 0 0 * 4 0 3 1 1 0 1 0 0 0 0 * ? 1 1 1 0 0 0 0 1 0 0 0 1 0 1***Creseis acicula*** 1 0 0 * 1 1 * 0 1 * * * * * * * * * 2 0 * * * 1 1 0 0 1 0 0 1 2 2 0 0 0 2 0 0 * 0 0 1 1 0 0 0 1 0 0 0 0 1 0 1***Creseis chierchiae*** 1 0 0 * 1 1 * 0 1 * * * * * * * * * 2 0 * * * 1 1 0 0 1 0 0 1 2 2 0 0 0 2 0 0 * 0 0 1 1 0 0 0 1 0 0 0 0 1 0 1***Creseis conica*** 1 0 0 * 1 1 * 0 1 * * * * * * * * * 2 0 * * * 1 1 0 0 1 0 0 1 2 2 0 0 0 2 0 0 * 0 0 1 1 0 0 0 1 0 0 0 0 1 0 1***Creseis virgula*** 1 0 0 * 1 1 * 0 1 * * * * * * * * * 2 0 * * * 1 1 0 0 1 0 0 1 2 2 0 0 0 2 0 0 * 0 0 1 1 0 0 0 1 0 0 0 0 1 0 1***Styliola subula*** 1 0 0 * 1 1 * 0 0 * * * * * * * * * 2 0 * * * 0 0 0 1 0 0 0 1 2 2 0 0 0 2 0 0 * 0 0 1 1 0 0 0 1 0 0 0 0 1 0 1***Hyalocylis striata*** 1 0 0 * 1 1 * 3 2 * * * * * * * * * 2 2 * * * 0 0 1 0 0 0 0 1 2 2 0 0 1 2 0 0 * 0 0 1 1 0 0 1 1 0 0 0 0 1 0 1***Cuvierina columnella*** 1 0 0 * 1 1 * 0 3 * * * * * * * * * 2 2 * * * 0 0 1 0 0 0 0 1 3 3 1 2 1 0 0 0 * 0 0 1 1 1 0 0 1 0 0 0 0 1 1 1***Cuvierina urceolaris*** 1 0 0 * 1 1 * 0 3 * * * * * * * * * 2 2 * * * 0 0 1 0 0 0 0 1 3 3 1 2 1 0 0 0 * 0 0 1 1 1 0 0 1 0 0 0 0 1 1 1***Cuvierina spoeli*** 1 0 0 * 1 1 * 0 3 * * * * * * * * * 2 2 * * * 0 0 1 0 0 0 0 1 3 3 1 2 1 0 0 0 * 0 0 1 1 1 0 0 1 0 0 0 0 1 1 1***Clio convexa*** 1 0 0 * 1 1 * 1 * 0 1 0 * 3 0 0 0 * 2 0 * * * 0 0 0 1 0 0 0 1 3 3 1 2 1 0 0 0 0 0 0 1 1 0 0 0 1 0 0 0 0 1 0 1***Clio cuspidata*** 1 0 0 * 1 1 * 1 * 0 0 1 * 3 0 1 0 * 2 0 * * * 0 0 0 1 0 0 0 1 3 3 1 2 1 0 0 1 0 0 0 1 1 0 0 0 1 0 0 0 0 1 0 1***Clio polita*** 1 0 0 * 1 1 * 1 * 0 1 0 * 0 0 0 0 * 2 0 * * * 2 0 0 1 * 4 0 3 3 3 1 2 1 0 0 0 0 0 1 1 1 0 0 0 1 ? 0 0 0 1 0 1***Clio pyramidata*** 1 0 0 * 1 1 * 1 * 0 0 0 * 3 0 0 0 * 2 0 * * * 0 0 0 1 0 0 0 1 3 3 1 2 1 1 0 0 0 0 0 1 1 0 0 0 1 0 0 0 0 1 0 1***Clio recurva*** 1 0 0 * 1 1 * 1 * 0 1 0 * 3 0 0 0 * 2 0 * * * 0 0 0 1 0 0 0 1 3 3 1 2 1 0 0 0 0 0 0 1 1 0 0 0 1 1 0 0 0 1 0 1***Clio chaptali*** 1 0 0 * 1 1 * 1 * 0 1 0 * ? ? 0 0 * 2 0 * * * 0 0 0 1 0 0 0 ? 3 3 1 2 1 0 0 0 0 0 0 1 1 0 0 0 1 1 0 0 0 1 0 1***Diacria trispinosa*** 1 0 0 * 1 1 * 2 * 1 0 1 0 5 0 0 1 1 2 1 * * * 0 0 0 1 0 0 0 1 3 3 1 2 0 0 0 0 0 0 0 1 1 0 0 0 1 0 0 0 0 1 0 1***Diacria rampali*** 1 0 0 * 1 1 * 2 * 1 0 1 0 5 0 0 1 1 2 1 * * * 0 0 0 1 0 0 0 1 3 3 1 2 0 0 0 0 0 0 0 1 1 0 0 0 1 0 0 0 0 1 0 1***Diacria gracilis*** 1 0 0 * 1 1 * 2 * 1 0 1 0 5 0 0 1 1 2 1 * * * 0 0 0 1 0 0 0 1 3 3 1 2 0 0 0 0 0 0 0 1 1 0 0 0 1 0 0 0 0 1 0 1***Diacria quadridentata*** 1 0 0 * 1 1 * 2 * 1 0 0 0 5 0 0 1 1 2 2 * * * 0 0 0 1 0 0 0 1 3 3 1 2 0 0 0 0 0 0 0 1 1 0 0 0 1 0 0 0 0 1 0 1***Cavolinia gibbosa*** 1 0 0 * 1 1 * 2 * 1 0 0 0 5/6 0 0 1 0 0 0 * * * 0 0 0 0 0 0 0 1 3 3 1 2 1 0 0 0 0 1 0 1 1 0 0 0 1 0 0 0 0 1 0 1***Cavolinia flava*** 1 0 0 * 1 1 * 2 * 1 0 0 0 7 0 0 1 0 0 0 * * * 0 0 0 0 0 0 0 1 3 3 1 2 1 0 0 0 0 1 0 1 1 0 0 0 1 0 0 0 0 1 0 1***Cavolinia gibboides*** 1 0 0 * 1 1 * 2 * 1 0 0 0 7 0 0 1 0 0 0 * * * 0 0 0 0 0 0 0 1 3 3 1 2 1 0 0 0 0 1 0 1 1 0 0 0 1 0 0 0 0 1 0 1***Cavolinia plana*** 1 0 0 * 1 1 * 2 * 1 0 0 0 5 0 0 1 0 0 0 * * * 0 0 0 0 0 0 0 1 3 3 1 2 1 0 0 0 0 1 0 1 1 0 0 0 1 0 0 0 0 1 0 1***Cavolinia globulosa*** 1 0 0 * 1 1 * 2 * 1 0 0 0 5 0 0 1 0 0 0 * * * 0 0 0 0 0 0 0 1 3 3 1 2 1 0 1 0 0 1 0 1 1 0 0 0 1 0 0 0 0 1 0 1***Cavolinia tridentata*** 1 0 0 * 1 1 * 2 * 1 0 0 0 3 1 0 1 0 0 0 * * * 0 0 0 0 0 0 0 1 3 3 1 2 1 0 0 0 1 1 0 1 1 0 0 0 1 0 0 0 0 1 0 1***Cavolinia uncinata*** 1 0 0 * 1 1 * 2 * 1 0 0 0 3 1 0 1 0 0 0 * * * 0 0 0 0 0 0 0 1 3 3 1 2 1 0 0 0 1 1 0 1 1 0 0 0 1 0 0 0 0 1 0 1***Cavolinia inflexa*** 1 0 0 * 1 1 * 2 * 1 0 0 0 3 0 0 1 0 0 0 * * * 0 0 0 0 0 0 0 1 3 3 1 2 1 0 0 0 0 1 0 1 1 0 0 0 1 0 0 0 0 1 0 1***Cavolinia labiata*** 1 0 0 * 1 1 * 2 * 1 0 0 0 1 0 0 1 0 0 0 * * * 0 0 0 0 0 0 0 1 3 3 1 2 1 0 0 0 0 1 0 1 1 0 0 0 1 0 0 0 0 1 0 1***Diacavolinia longirostris*** 1 0 0 * 1 1 * 2 * 1 0 0 1 3 0 0 2 0 1 0 * * * 0 0 0 0 0 0 0 1 3 3 1 2 1 0 1 0 0 1 0 1 1 0 0 0 1 0 1 0 0 1 0 1***Peraclis apicifulva*** 1 0 0 * 0 0 1 * * * * * * * * * * * 0 * * * * 2 0 0 0 * 1 1 3 0 1 0 1 * 0 0 0 * 2 1 1 1 0 1 0 1 0 0 0 1 1 0 0***Peraclis bispinosa*** 1 0 0 * 0 0 1 * * * * * * * * * * * 0 * * * * 2 0 0 0 * 1 1 3 0 1 0 1 * 0 0 0 * 2 1 1 1 0 1 0 1 0 0 0 1 1 0 0***Peraclis depressa*** 1 0 0 * 0 0 1 * * * * * * * * * * * 0 * * * * 2 0 0 0 * 1 1 3 0 1 0 1 * 0 0 0 * 2 1 1 1 0 1 0 1 0 0 0 1 1 0 0***Peraclis moluccensis*** 1 0 0 * 0 0 1 * * * * * * * * * * * 0 * * * * 2 0 0 0 * 1 1 3 0 1 0 1 * 0 0 0 * 2 1 1 1 0 1 0 1 0 0 0 1 1 0 0***Peraclis reticulata*** 1 0 0 * 0 0 1 * * * * * * * * * * * 0 * * * * 2 0 0 0 * 1 1 3 0 1 0 1 * 0 0 0 * 2 1 1 1 0 1 0 1 0 0 0 1 1 0 0***Peraclis triacantha*** 1 0 0 * 0 0 1 * * * * * * * * * * * 0 * * * * 2 0 0 0 * 1 1 3 0 1 0 1 * 0 0 0 * 2 1 1 1 0 1 0 1 0 0 0 1 1 0 0***Peraclis valdiviae*** 1 0 0 * 0 0 1 * * * * * * * * * * * 0 * * * * 2 0 0 0 * 1 1 3 0 1 0 1 * 0 0 0 * 2 1 1 1 0 1 0 1 0 0 0 1 1 0 0***Cymbulia peroni*** 1 1 1 1 * * * * * * * * * * * * * * * * 1 1 0 * * * * * 1 1 2 0 0 * 0 0 ? 0 0 * 3 ? 1 1 0 0 0 1 0 0 0 ? 1 0 0***Cymbulia parvidentata*** 1 1 1 1 * * * * * * * * * * * * * * * * 1 1 0 * * * * * 1 1 2 0 0 * 0 0 ? 0 0 * 3 ? 1 1 0 0 0 1 0 0 0 ? 1 0 0***Cymbulia sibogae*** 1 1 1 1 * * * * * * * * * * * * * * * * 1 1 0 * * * * * 1 1 2 0 0 * 0 0 ? 0 0 * 3 ? 1 1 0 0 0 1 0 0 0 ? 1 0 0***Gleba cordata*** 1 1 1 0 * * * * * * * * * * * * * * * * 1 0 1 * * * * * 3 1 2 ? ? ? ? ? ? 0 0 * ? ? 0 0 * * * * 0 0 0 0 1 0 0***Corolla chrysosticta*** 1 1 1 0 * * * * * * * * * * * * * * * * 1 0 1 * * * * * 2 1 2 ? ? ? ? ? ? 0 0 * ? ? 0 0 * * * * 0 0 0 0 1 0 0***Corolla cupula*** 1 1 1 0 * * * * * * * * * * * * * * * * 1 0 1 * * * * * 2 1 2 ? ? ? ? ? ? 0 0 * ? ? 0 0 * * * * 0 0 0 ? 1 0 0***Corolla calceola*** 1 1 1 0 * * * * * * * * * * * * * * * * 1 0 1 * * * * * 2 1 2 ? ? ? ? ? ? 0 0 * ? ? 0 0 * * * * 0 0 0 ? 1 0 0***Corolla intermedia*** 1 1 1 0 * * * * * * * * * * * * * * * * 1 0 1 * * * * * 2 1 2 ? ? ? ? ? ? 0 0 * ? ? 0 0 * * * * 0 0 0 ? 1 0 0***Corolla ovata*** 1 1 1 0 * * * * * * * * * * * * * * * * 1 0 1 * * * * * 2 1 2 ? ? ? ? ? ? 0 0 * ? ? 0 0 * * * * 0 0 0 ? 1 0 0***Corolla spectabilis*** 1 1 1 0 * * * * * * * * * * * * * * * * 1 0 1 * * * * * 2 1 2 ? ? ? ? ? ? 0 0 * ? ? 0 0 * * * * 0 0 0 ? 1 0 0***Desmopterus papilio*** 0 * * * * * * * * * * * * * * * * * * * 0 0 0 * * * * * 4 ? ? ? ? ? ? ? ? ? ? * ? ? 0 1 ? ? ? ? ? ? ? ? ? ? ?***Desmopterus gardinieri*** 0 * * * * * * * * * * * * * * * * * * * 0 0 0 * * * * * 4 ? ? ? ? ? ? ? ? ? ? * ? ? 0 1 ? ? ? ? ? ? ? ? ? ? ?***Desmopterus pacificus*** 0 * * * * * * * * * * * * * * * * * * * 0 0 0 * * * * * 4 ? ? ? ? ? ? ? ? ? ? * ? ? 0 1 ? ? ? ? ? ? ? ? ? ? ?
